# Confined growth of UiO-66 into ultrahigh-loading membranes for efficient hexane isomer separation†

**DOI:** 10.1039/d5sc04212g

**Published:** 2025-07-19

**Authors:** Pan-Pan Zhang, Jing-Ran Yu, Jia-Yu Ding, Wei-Hai Lin, Zhen Chen, Wei Shao, Shu-Chang Wang, Yi-Le Chen, Yi Li, Qi-Han Gong, Ming Xue, Xiao-Ming Chen

**Affiliations:** a School of Chemical Engineering and Technology, Southern Marine Science and Engineering Guangdong Laboratory (Zhuhai), MOE Key Laboratory of Bioinorganic and Synthetic Chemistry, GBRCE for Functional Molecular Engineering, School of Chemistry, IGCME, Zhuhai Key Laboratory of Optoelectronic Functional Materials and Membrane Technology, Sun Yat-sen University Guangzhou 510275 China xueming5@mail.sysu.edu.cn liyi266@mail.sysu.edu.cn; b Fundamental Science & Advanced Technology Lab, PetroChina Petrochemical Research Institute Beijing 102200 China gongqihan@petrochina.com.cn

## Abstract

The efficient separation of hexane isomers is a crucial process in the petrochemical industry. Mixed-matrix membranes (MMMs) hold tremendous potential for hexane isomer separation. However, maintaining their continuity at high filler loading remains a substantial challenge. Here, UiO-66/PP mixed-matrix membranes are fabricated *via* an *in situ* confined growth synthesis strategy that achieves an exceptional filler loading of 72.9 wt%. The resulting UiO-66/PP-(96) membrane maintains structural continuity while effectively discriminating linear and mono-branched hexane isomers from their di-branched counterparts, exhibiting a flux of 473.5 g m^−2^ h^−1^ and a separation factor of 4.53 for *n*-hexane/2,2-dimethylbutane mixtures. Remarkably, this membrane enriches *n*-hexane content from 50.0 wt% in the feed to 81.9 wt% in the permeate through a single processing stage, while retaining robust performance across various hexane isomer combinations. These characteristics highlight its potential for extracting linear alkanes to enhance the gasoline research octane number (RON). Molecular dynamics (MD) simulations corroborate these findings, revealing faster transport kinetics for *n*-hexane compared to branched isomers. This straightforward synthesis approach presented herein significantly broadens the avenues for the advancement of MOF-based mixed-matrix membranes in petrochemical separation applications.

## Abstract 2

High stable membranes are fabricated by an *in situ* confined growth strategy of UiO-66 NPs in the nanochannels of PP under ambient conditions, with filler loading up to 72.9 wt%, exhibiting exceptional separation performance for hexane isomers.

## Graphical abstract

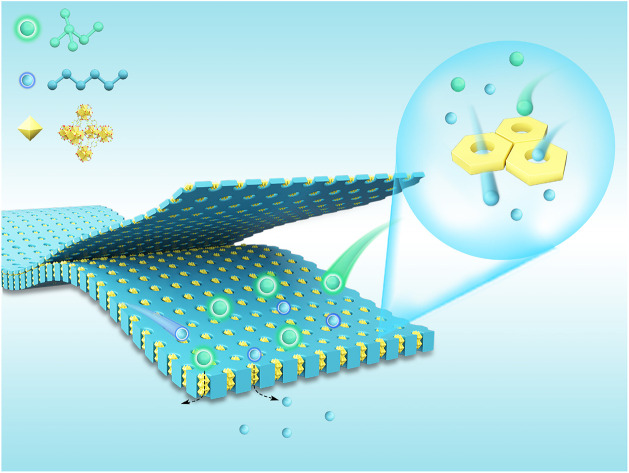

## Introduction

Light naphtha, a valuable refinery byproduct, contributes to improved oil product quality due to its beneficial low-temperature properties and combustion characteristics. Primarily composed of C_6_ hydrocarbons, it includes linear and branched isomers. Linear and mono-branched hexane isomers are suitable as feedstock for cracked olefin (ethylene), while di-branched hexane isomers are suitable for use as a blending component in high-quality gasolines. The Research Octane Number (RON), a measure of fuel anti-knock resistance, is indicative of its resistance to detonation in internal combustion engines. Significantly, the di-branched hexane isomers, 2,3-dimethylbutane (23DMB) and 2,2-dimethylbutane (22DMB), display high RON values of 105 and 94, respectively, whereas the linear *n*-hexane (*n*-Hex) exhibits a low RON of 30.^1–3^ In response to market shifts towards lead-free gasoline and regulatory restrictions on additives, the separation of linear and mono-branched hexane isomers from the di-branched isomers is essential to meet these standards.^4,5^ Considerable efforts have been dedicated to the separation of the di-branched isomers from the hexane isomers.^6–8^ Traditional thermal-driven separation techniques, such as distillation, drying, and volatilization, are high-energy-consuming and equipment-intensive processes.^9,10^

Membrane separation is considered a promising alternative to traditional distillation, offering advantages such as low energy consumption, environmental sustainability, and pollution-free operation.^11–15^ Polymer membranes are typically constrained by the classic compromise between permeability and selectivity, a well-known challenge in membrane science.^16,17^ In contrast, microporous inorganic membranes are extensively employed in molecular separations due to their well-defined pore architecture and robust stability. However, their large-scale application remains limited by the lack of facile and reproducible fabrication methods.^18–21^ To address these challenges, mixed-matrix membranes (MMMs) have been developed, combining the advantages of inorganic fillers and organic polymers.^22,23^ However, achieving an even dispersion of fillers in polymer matrices and controlling interfacial compatibility while ensuring a defect-free interface at high filler loadings (≥40 wt%) remains a significant challenge.^24–26^ Recently, Jin *et al.* employed a solid–solvent process to fabricate an MMM with 80% high metal–organic framework (MOF) loading, demonstrating inorganic-like membrane separation performance and the H_2_/CO_2_ separation performance with H_2_/CO_2_ selectivity 1–2 orders of magnitude higher than state-of-the-art membranes.^27^ A critical issue at high filler loadings is the formation of non-selective pores, which leads to reduced separation performance. To overcome this, *in situ* confined growth of porous fillers within the macropores of a polymer substrate has been proposed. This approach aims to create a novel MMM where fillers constitute the main matrix and serve as the predominant phase, the penetrating pores formed between the fillers are expected to provide ultra-fast transport channels for molecules, thereby potentially surpassing the upper bound of membrane permeability and selectivity and optimizing the membrane separation performance.

MOFs are built from metal ions/clusters and organic ligands through coordination interactions to form periodically open and extended structures,^28–30^ which feature designability of building blocks, uniform aperture size, diverse modifiability and high surface area. These properties endow MOFs with a wide range of applications, such as gas separation, especially as adsorption media,^31–35^ so MOFs have made significant progress in the separation of hydrocarbons such as C_2_H_2_/C_2_H_4_/C_2_H_6_, C_3_H_6_/C_3_H_8_, butene isomers, xylene isomers,^36–40^*etc.*, and also have great potential for the separation of alkane isomers. MOF-508 was the first reported example of alkane isomer separation by Chen *et al.*, achieved through fine pore size and shape matching, thus leading to selectivity.^41^ Recently, many other MOFs with different structures have been reported in hexane isomer separation, while membrane separation of hexane isomers is rarely reported.^42–47^

**Scheme 1:**
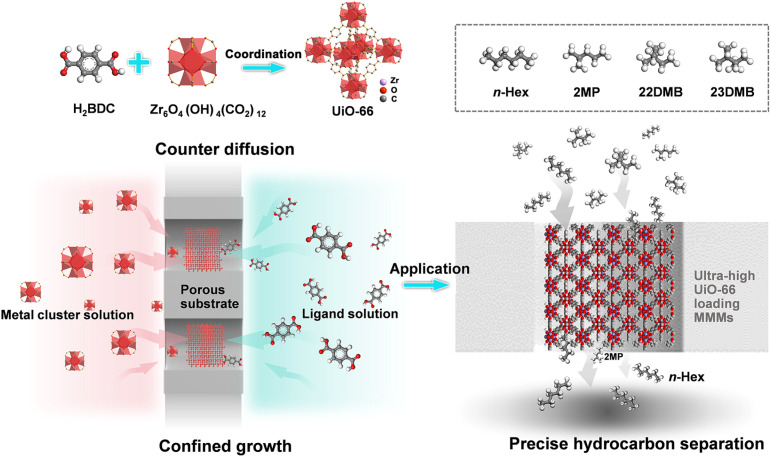
Schematic illustration of fabricating a UiO-66 membrane in a confined space and its application in hexane isomer separation.

In this work, a new strategy was proposed, termed the *in situ* confined assembly strategy, for fabricating UiO-66/PP mixed-matrix membranes (MMMs) *via* counter-diffusion of pre-synthesized metal cluster precursors and organic ligand solutions (Scheme 1). Unlike conventional methods, this approach enables the growth and dense packing of UiO-66 nanoparticles (NPs)^48,49^ within the substrate macropores rather than on its surface, achieved by precisely controlled diffusion kinetics through the porous network. The crystal growth time was carefully controlled to ensure tight packing of UiO-66 within the pores of the substrate, thereby inhibiting the formation of defects such as inter-crystalline and lattice defects. The obtained UiO-66/PP-(96) membrane with an ultrahigh UiO-66 loading of 72.9 wt% demonstrated excellent separation performance for hexane isomers. Such mixed-matrix membranes, with tightly packed MOF NPs in the pores of organic polymers serving as the main phase, can be expected to solve the dilemma between flux and selectivity, and have a bright prospect for hexane isomer separation.

## Results and discussion

**Fig. 1:**
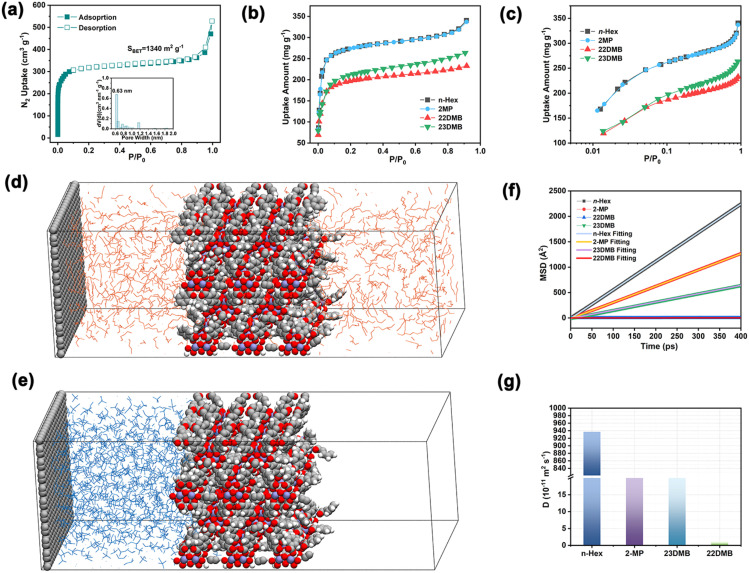
(a) N_2_ adsorption–desorption isotherm measured at 77 K for UiO-66 and the pore size distribution; adsorption isotherms of hexane isomers: (b) linear and (c) logarithmic scale of *n*-Hex, 2MP, 23DMB and 22DMB for UiO-66. The diffusion behaviors of (d) *n*-Hex (orange) and (e) 22DMB (blue) on UiO-66 obtained by MD simulation; (f) MSD plots of *n*-Hex, 2MP, 23DMB and 22DMB in UiO-66; (g) self–diffusion coefficient of *n*-Hex, 2MP, 23DMB and 22DMB in the UiO-66 material.

UiO-66 NPs were synthesized at room temperature, and nitrogen adsorption isotherms revealed a characteristic Type I profile, confirming the microporous structure. The Brunauer–Emmett–Teller (BET) surface area was calculated to be 1340 m^2^ g^−1^, and the pore size distribution curves were obtained using nonlocal density functional theory (NL-DFT). As can be seen from Fig. 1a, the main pore sizes are concentrated at 0.63 nm, and the total pore volume of UiO-66 is 0.524 cm^3^ g^−1^. The amount of adsorption showing an upward trend at high pressure is a result of the interparticle voids of UiO-66 aggregates. Scanning electron microscopy (SEM) confirmed a near-spherical morphology with an average crystal size of 130 nm (Fig. S1a†). Dynamic light scattering (DLS) analysis in methanol yielded a particle size of 128 ± 4 nm (Fig. S1b†), closely matching SEM observations. This controlled nanoscale dimension results from the slow nucleation and growth under ambient conditions, which facilitates confined assembly in the macropores of polymer matrices.

Single-component adsorption isotherms of *n*-Hex, 2MP, 22DMB and 23DMB on UiO-66 were measured at 298 K (Fig. 1b and c). UiO-66 exhibits distinct adsorption preferences: at low pressures, 22DMB shows the slowest uptake, with equilibrium capacities following the sequence *n*-Hex (340.5 mg g^−1^) > 2MP (330.7 mg g^−1^) > 23DMB (263.6 mg g^−1^) > 22DMB (232.5 mg g^−1^). This performance combines high surface area and exceptional *n*-Hex uptake, surpassing most recently reported MOFs such as NU-80-LP (220 mg g^−1^) and ZIF-8 (236 mg g^−1^) (Table S1†). Critically, adsorption capacities for linear and mono-branched isomers significantly surpass those of di-branched isomers. This trend inversely correlates with molecular dynamic diameters: 22DMB (6.2 Å) > 23DMB (5.6 Å) > 2-MP (5.0 Å) > *n*-Hex (4.3 Å). The interaction between the hexane isomer and the UiO-66 framework is determined by isosteric heat of adsorption (*Q*_st_), which was calculated according to the adsorption curves of 298 K and 323 K. The obtained *Q*_st_ of *n*-Hex and 2MP is higher than that of di-branched isomers (Fig. S2b†), confirming the preferential adsorption toward linear and mono-branched hexane isomers. The marked isosteric heats of adsorption (*Q*_st_) differences (12.8 kJ mol^−1^) between *n*-Hex and 22DMB suggest an adsorption thermodynamics mechanism, illustrating van der Waals interactions between alkyl chains and the terephthalate linkers of UiO-66. Molecular dynamics (MD) simulations of the directed diffusion of the four different hexane isomers in UiO-66 were carried out using the Gromacs program suite, with a hybridized force field composed of the TraPPE-UA force field and UFF4MOFII force field. The UFF parameters^50,51^ were selected based on their proven accuracy in modeling MOFs, as demonstrated in studies of adsorption and diffusion.^52^ TraPPE-UA, while simplified with four pseudo-atom types, has been extensively validated for alkane simulations, achieving a balance between computational efficiency and accuracy in reproducing thermodynamic and transport properties.^53^ The mean squared displacements (MSDs) of *n*-Hex, 2-MP, 23DMB, and 22DMB in UiO-66 are calculated. The analysis revealed significantly enhanced diffusion of *n*-Hex through the channels of UiO-66, whereas 22DMB exhibited negligible displacement over the 100 ns simulation period (Fig. 1d and e), additional snapshots for 2-MP and 23DMB can be seen in Fig. S3.† Self-diffusion coefficients, determined from the linear regime of the MSD plots, demonstrated a pronounced size-dependent trend: *n*-Hex (9.4 × 10^−9^ m^2^ s^−1^) > 2MP (5.3 × 10^−9^ m^2^ s^−1^)> 23DMB (2.7 × 10^−9^ m^2^ s^−1^) > 22DMB (1 × 10^−11^ m^2^ s^−1^) separately. Notably, *n*-Hex displayed a diffusion coefficient approximately 940-fold greater than that of 22DMB, highlighting the remarkable capacity of UiO-66 for size-selective molecular transport. To assess sensitivity to initial conditions, control simulations with randomized initial configurations of *n*-Hex and 22DMB were performed. Despite the variations, the calculated diffusion coefficients showed minimal deviation (Fig. S4†). The results indicate that *n*-Hex can permeate the pore wall preferentially; it was difficult for 23DMB and 22DMB to permeate through the pore windows of UiO-66. This combined evidence from adsorption studies and molecular dynamics simulations establishes UiO-66 as a promising candidate for the selective separation of hexane isomers.

**Fig. 2:**
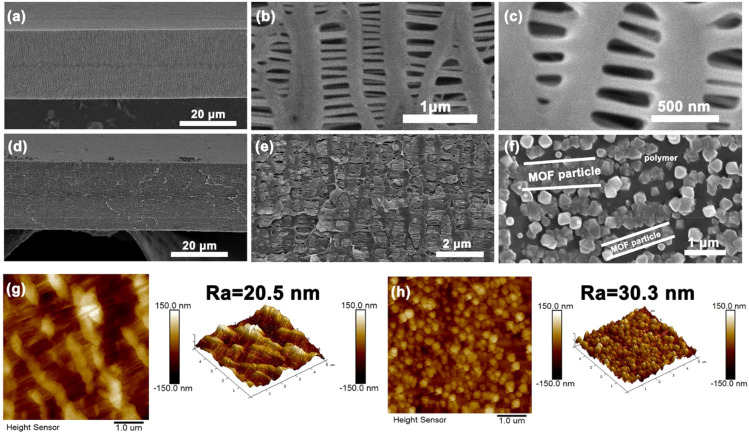
(a) Cross-section SEM image of neat PP; (b) enlarged image of (a); (c) top view image of neat PP; (d) cross-section SEM image of UiO-66/PP-(96); (e) enlarged image of (d); (f) top view image of UiO-66/PP-(96); 2D AFM surface morphology (left) and 3D surface roughness (right) of (g) PP and (h) UiO-66/PP-(96), respectively.

The above adsorption results prompt us to evaluate UiO-66-based membranes for practical hexane isomer separation. Mixed-matrix membranes (MMMs) are typically produced using a solution mixing approach; however, issues such as filler agglomeration, sedimentation, and interfacial defects between the filler and polymer matrix may occur during the solvent evaporation process, particularly when the filler loading is high. To avoid the above problems, we developed an alternative *in situ* self-assembly approach. This strategy employs opposite-diffusion of precursors to grow UiO-66 crystals directly within the macropores of polypropylene (PP) substrates under ambient conditions. As illustrated in Fig. 2a–c, the neat PP membrane exhibits a porous architecture with heterogeneous, strip-like pores that facilitate precursor diffusion and subsequent coordination assembly. Notably, crystal growth occurs preferentially within the macropores rather than at the membrane surface. Systematic investigation of assembly time dependence revealed progressive pore filling evolution (Fig. S5a–d†), where limited UiO-66 incorporation was observed after 24 h of growth (UiO-66/PP-(24), Fig. S5c†). Followed by near-complete pore occupation at 72 h (UiO-66/PP-(72)) with only minor residual voids (Fig. S5d†), ultimately achieving continuous membrane formation through full pore packing after 96 h of confined growth (UiO-66/PP-(96)). The gradual filling kinetics likely reflect the slow diffusion of metal clusters and ligands through the PP macropores. This confined growth approach successfully avoids the pitfalls of traditional MMM fabrication while enabling precise control over membrane morphology. The SEM image of UiO-66/PP-(96) in Fig. 2d shows a membrane thickness of 25 μm, which is consistent with neat PP. From the enlarged view of the cross-section in Fig. 2e, two phases of UiO-66 and PP were observed. UiO-66 NPs were packed densely in the PP pores, without visible agglomeration or cracks, and the particle size is around 125 nm, similar to the above-mentioned UiO-66 powder. Notably, when the PP substrate pores are fully packed with UiO-66 NPs, further diffusion of metal clusters and organic ligands is sterically hindered, preventing overgrowth and associated stress-induced defects, as evidenced by the defect-free morphology of UiO-66/PP-(120), which exhibits comparable performance to UiO-66/PP-(96) (Fig. S6†). TEM analysis (Fig. S7†) further verified the defect-free interface and dense packing of UiO-66 within PP pores. In contrast, conventional dip-coating methods (UiO-66/PP-(24_dc_), UiO-66/PP-(72_dc_), and UiO-66/PP-(96_dc_)) produced discontinuous MOF layers owing to low heterogeneous nucleation density within the pores of the PP substrate (Fig. S8†). AFM images in Fig. 2g reveal that the untreated PP membrane features a smooth surface with an average roughness of 20.5 nm. UiO-66/PP membranes develop progressively rougher morphology with increasing reaction time (Fig. S9†), reaching *R*_a_ = 30.3 nm for UiO-66/PP-(96) (Fig. 2h). The modest increase is attributable to pore packing rather than surface polycrystal formation, as confirmed by the roughness scale being substantially smaller than NP dimensions. This phenomenon is consistent with the image in Fig. 2f. Energy-dispersive X-ray (EDX) spectroscopy revealed uniform distributions of oxygen (O) and zirconium (Zr) in the membranes, in which the elemental concentrations of UiO-66/PP-(96) are much denser (Fig. S10†).

**Fig. 3:**
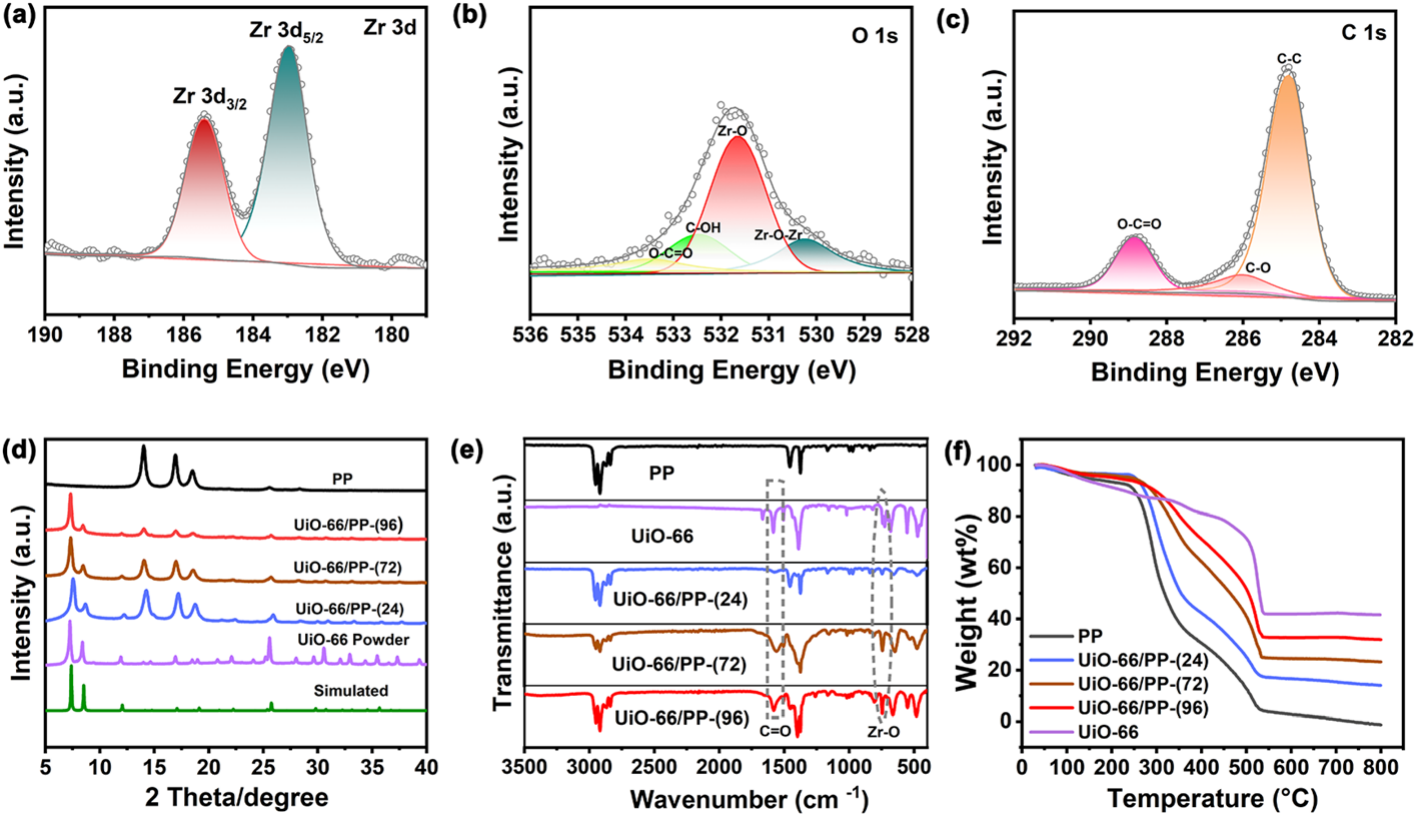
High-resolution XPS spectra for UiO-66/PP-(96): (a) Zr 3d; (b) O 1s; (c) C 1s; (d) PXRD patterns; (e) FT-IR spectra; (f) TGA curves for UiO-66 powder, neat PP and UiO-66/PP membranes.

X-ray photoelectron spectroscopy (XPS) was employed to investigate the chemical compositions and states of elements in the UiO-66/PP membrane. As illustrated in the Fig. 3a–c, high-resolution spectroscopy of C 1s, O 1s and Zr 3d for UiO-66/PP-(96) were fitted to analyze the bond states. The C 1s spectrum was fitted into peaks corresponding to C–C (284.8 eV), C–O (286.1 eV), and C=O (288.9 eV) bonds. The O 1s spectrum displayed peaks assigned to Zr–O–Zr (530.2 eV), Zr–O (531.8 eV), C–OH (532.5 eV), and O–C=O (533.5 eV) species. Additionally, the Zr 3d spectrum exhibited a bimodal split, with peaks attributed to Zr 3d_5/2_ (182.7 eV) and Zr 3d_3/2_ (185.1 eV) of ZrO_2_. These data are consistent with those of UiO-66 (ref. 54), and the binding energy has not been significantly shifted, confirming structural integrity within the PP matrix (Fig. S11†). Comparative XPS studies of neat PP and UiO-66/PP membranes with varying growth durations further validated these findings (Fig. S12–14†). Powder X-ray diffraction (PXRD) analysis confirmed the crystalline structure of both synthesized UiO-66 NPs and UiO-66/PP membranes (Fig. 3d). The experimental pattern of UiO-66 powder matches well with that of the simulated one, confirming phase purity, and the characteristic diffraction peaks appearing at 2*θ* = 7.29°, 8.44° and 25.68° correspond to the (111), (200), (200) and (600) lattice planes, respectively.^54^ Time-dependent studies revealed that all UiO-66/PP membranes retained the crystalline signature of UiO-66, confirming structural preservation within the polymer matrix. Notably, the diffraction peak intensities showed a progressive enhancement with increasing growth time, directly correlating with the degree of crystalline packing within the PP substrate. Fourier transform infrared (FT-IR) spectroscopy further verified UiO-66 incorporation within the membrane. As depicted in Fig. 3e, the adsorption band centered at 745 cm^−1^ (Zr–O) in the membrane indicates the coordination of Zr clusters with the carboxylate ligands, and the those at 1578 cm^−1^ and 1397 cm^−1^ are then attributed to the symmetric and asymmetric stretching vibration peaks of the carboxylate group in UiO-66 (ref. 54), respectively. Thermogravimetric analysis (TGA) subsequently quantified the MOF loadings across the UiO-66/PP membrane series (Fig. 3f). The derivative thermogravimetry (DTG) curves for the PP membrane exhibit major weight losses at 292 °C and 505 °C, corresponding to its thermal degradation (Fig. S15†). For pure UiO-66, three distinct weight loss stages are observed: the first at 100 °C, attributed to the removal of physically adsorbed water molecules; the weight loss observed around 374 °C and 525 °C is due to the framework decomposition. The maximum weight loss for UiO-66/PP progressively shifts toward that of pure UiO-66 as the filler content increases. This behavior suggests strong interfacial interactions and uniform dispersion of UiO-66 within the PP matrix, which restricts polymer chain mobility and delays thermal degradation. The TGA data quantitatively confirmed UiO-66 loadings of 22.5 wt%, 52.3 wt%, and 72.9 wt% for the respective membranes, providing insights into the precise composition of each membrane. Further supported by ICP-AES measurements (Table S2†). High loadings of UiO-66 create a molecular percolation highway, which facilitates the rapid diffusion of hexane isomers and enhances separation performance.

The intrinsic structural benefits of UiO-66 and the simplicity of the membrane fabrication process prompted us to evaluate the potential of UiO-66/PP mixed-matrix membranes for their efficiency in actual hexane isomer separation. Prior to the pervaporation test, N_2_ adsorption–desorption measurements at 77 K for both the pristine PP membrane and UiO-66/PP-(96) MMM were conducted to quantify the pore size distribution. The PP substrate exhibits negligible N_2_ uptake, whereas UiO-66/PP-(96) shows a Type I isotherm characteristic of microporous materials, with a micropore volume of 0.345 cm^3^ g^−1^ and pore size distribution peaks at 0.64 nm and 1.17 nm (Fig. S16†). These values align precisely with the intrinsic pore structure of UiO-66 NPs, confirming that UiO-66 dominates the pore architecture of the MMM. CO_2_/CH_4_ gas mixture separation was conducted to further evaluate the quality of the representative membranes (PP, UiO-66/PP-(24) and UiO-66/PP-(96)). As shown in Fig. S17,† the CO_2_ permeance for the PP membrane is 639.5 × 10^−8^ mol m^−2^ s^−1^ Pa^−1^, while the CO_2_/CH_4_ selectivity is less than 1, far lower than that of Knudsen diffusion, indicating poor separation performance. In contrast, UiO-66/PP-(96) exhibited a substantially reduced CO_2_ permeance of 9.67 × 10^−8^ mol m^−2^ s^−1^ Pa^−1^ and a markedly improved CO_2_/CH_4_ selectivity of 18.6, exceeding the Knudsen diffusion value and confirming high quality of the membrane.

Subsequently, pervaporation experiments using home-made equipment for the separation of linear and branched hexane isomers were performed at room temperature. Few studies in the literature have been conducted on MOF-based membranes for hexane isomer separation, implying that this topic remains an area of challenge. Our UiO-66/PP-(96) membrane demonstrates exceptional performance. The measured flux values for *n*-Hex, 2MP, 23DMB and 22DMB were 401.9, 371.3, 166.0 and 91.7 g m^−2^ h^−1^ respectively (Table S3†). Single-component permeation tests yielded an ideal separation factor of 4.38 for *n*-Hex over 22DMB. The great selectivity separation factor for UiO-66/PP-(96) suggests preferential permeation through UiO-66 pores, presumably caused by molecular affinity and diffusion freedom toward *n*-Hex rather than 22DMB. To prove this synergistic effect, the permeance (*P*) was deconvoluted into solubility (*S*) and diffusivity (*D*) (Table S4†). The solubility coefficients, determined by fitting adsorption isotherms (Fig. S18–21†), reveal that the UiO-66/PP-(96) membrane exhibits stronger adsorption affinity for linear and mono-branched isomers compared to di-branched isomers. This preferential adsorption leads to more compact packing of linear and mono-branched isomers within UiO-66 pores, resulting in higher solubility selectivity. The derived diffusion coefficients further corroborate the molecular dynamics trends: *n*-Hex and 2MP diffuse faster (*D*_*n*-Hex_ = 9.69 × 10^−12^ m^2^ s^−1^; *D*_2MP_ = 9.45 × 10^−12^ m^2^ s^−1^) than 23DMB and 22DMB (*D*_23DMB_ = 7.21 × 10^−12^ m^2^ s^−1^; *D*_22DMB_ = 6.95 × 10^−12^ m^2^ s^−1^) (Table S4†). Notably, the diffusion selectivity (*D*_*n*-Hex_/*D*_22DMB_ = 1.39) is significantly lower than the solubility selectivity (*S*_*n*-Hex_/_22DMB_ = 3.14), indicating that adsorption thermodynamics dominate the overall membrane selectivity (Table S5†).

**Fig. 4:**
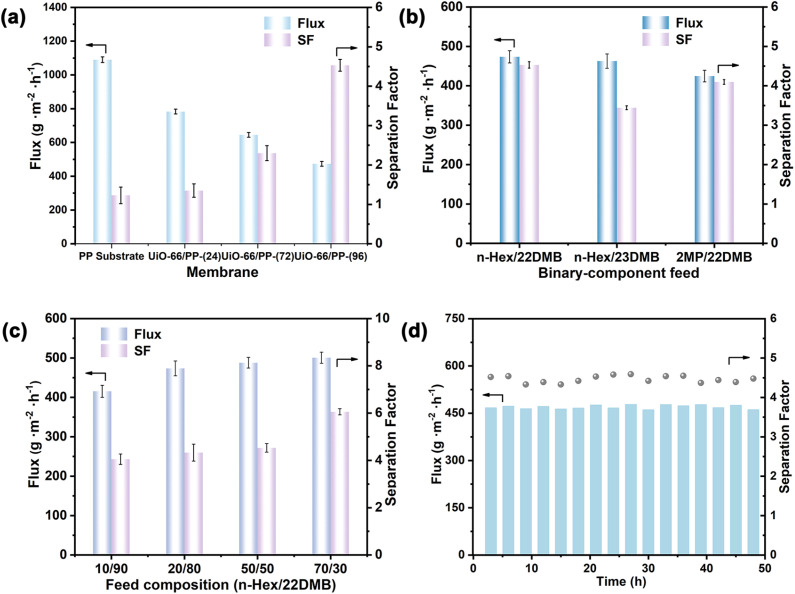
(a) Separation performance of membranes with different UiO-66 loadings for binary-component hexane isomers; flux and separation factor of the UiO-66/PP-(96) membrane for (b) different binary hexane isomers and (c) *n*-Hex/22DMB mixtures with different *n*-Hex concentrations; (d) long-term testing of the UiO-66/PP-(96) membrane.

Besides the single-component test, binary-component separation was performed on the representative membrane. Fig. 4a shows that the membranes achieved exceptional flux and selectivity for *n*-Hex/22DMB. While the neat PP membrane demonstrated an initial flux of 1090 g m^−2^ h^−1^, it showed poor separation performance with an *n*-Hex/22DMB separation factor of only 1.23. The incorporation of UiO-66 nanoparticles through *in situ* growth within the PP substrate significantly altered these properties, forming structurally continuous mixed-matrix membranes. Quantitatively, with increasing UiO-66 content from 0% to 72.9 wt%, we observed a decrease in flux accompanied by a substantial enhancement in selectivity, reaching an optimal *n*-Hex/22DMB separation factor of 4.55 at the highest UiO-66 loading. Meanwhile, the high PSI value of 1671.6 (Table S6†) for *n*-Hex/22DMB reflects an optimal trade-off between permeation flux and separation factor, demonstrating the critical role of UiO-66 in enhancing the separation factor of *n*-Hex over 22DMB. This phenomenon stems from the adsorption affinity of UiO-66, which optimizes the molecular separation effect when sufficient UiO-66 NP loading is achieved. Fig. 4b exhibits the separation of diverse combinations of hexane isomers. The UiO-66/PP-(96) membrane showed a total flux of 482.6 g m^−2^ h^−1^ with a separation factor of 3.44 for 50/50 *n*-Hex/23DMB mixtures, and achieved 424.6 g m^−2^ h^−1^ flux with 4.1 selectivity for 2MP/22DMB mixtures. In contrast, conventional methods such as dip-coating (UiO-66/PP-(24_dc_), UiO-66/PP-(72_dc_), and UiO-66/PP-(96_dc_)) resulted in lower separation performance due to the discontinuous MOF layers (Table S7†). The *in situ* confined growth strategy leverages bidirectional diffusion of Zr clusters and terephthalic acid within the pores of the PP substrate, enabling spatially controlled nucleation and dense stacking of UiO-66 NPs. The superiority of our method lies in three key innovations: (1) pore confinement: restricting MOF growth to PP pores minimizes interfacial stress, ensuring mechanical stability even at high filler loadings; (2) self-limiting diffusion: bidirectional reagent diffusion prevents overgrowth and pore blockage, maintaining hierarchical transport pathways. (3) Simplicity: the process avoids toxic solvents and complex post-treatments, enabling continuous membrane fabrication. These results highlight the membrane potential for separating linear and mono-branched alkanes from di-branched isomers, particularly for high-RON gasoline enrichment.

Fig. 4c illustrates the dependence of permeation flux and *n*-Hex/22DMB separation factor on feed concentration for the UiO-66/PP-(96) membrane. The membrane demonstrates consistent selectivity for *n*-Hex across both dilute and concentrated feeds, maintaining an average separation factor of 4.05 for 10/90 *n*-Hex/22DMB mixtures. This sustained selectivity arises from preferential permeation of *n*-Hex through the pores of UiO-66 instead of the voids of PP. Notably, the separation factor increases with rising *n*-Hex concentration, a phenomenon explained by two synergistic mechanisms: (1) higher *n*-Hex feed concentrations promote membrane adsorption, thereby increasing the solubility coefficient and enhancing permeation; (2) elevated saturated vapor pressure provides greater driving force for molecular diffusion through the membrane. These concentration-dependent effects collectively enhance the membrane separation efficiency while maintaining its structural integrity.

From a practical application perspective, we evaluated the long-term performance of a representative UiO-66/PP-(96) membrane for *n*-Hex/22DMB (50/50) separation. The membrane demonstrated excellent stability over 48 h, maintaining a consistent flux of 469.2 ± 6.0 g m^−2^ h^−1^ and an average *n*-Hex/22DMB separation factor of 4.46 (Fig. 4d). Post-stability XRD analysis confirmed the preserved crystallinity of UiO-66 in the membrane (Fig. S22†). Compared with the state-of-the-art zeolite and MOF membranes (Table S8†), UiO-66/PP-(96) achieves a superior flux while maintaining a competitive separation factor for *n*-Hex/22DMB. Furthermore, the membrane exhibits remarkable resistance to feed impurities, as demonstrated by tests using a challenging ternary mixture (*n*-Hex/22DMB/toluene/*m*-xylene = 45 : 45 : 5 : 5 vol%), which yielded comparable performance (Fig. S23†) to the pure binary system. This represents a significant improvement over our previous UiO-66/PIM-1-(20) membrane, whose maximum selectivity of 3.14 was limited by lower filler loading capacity. Control experiments have initiated preliminary investigations using the confined counter-diffusion method in other MOF/polymer combinations. For instance, we fabricated a ZIF-8/PP-(96) membrane using deionized water as solvent and observed a separation factor of 1.8 for *n*-Hex/22DMB, significantly lower than that for UiO-66/PP-(96); similarly, substituting the PP substrate with a microfiltration PP membrane resulted in discontinuous UiO-66 growth due to mismatched pore size and solvent affinity (Fig. S24 and Table S9†). The results highlight that the success of our method depends on careful alignment of substrate pore size, solvent compatibility, and MOF nucleation kinetics. The superior performance of UiO-66/PP-(96) arises from two synergistic factors: (1) pore size matching: the pores of the substrate allow bidirectional diffusion of Zr clusters and ligands while restricting MOF growth within the nanochannels, ensuring interfacial adhesion; (2) solvent hydrophobicity: the non-polar PP matrix minimizes solvent-induced swelling during MOF synthesis, preserving structural integrity.

**Fig. 5:**
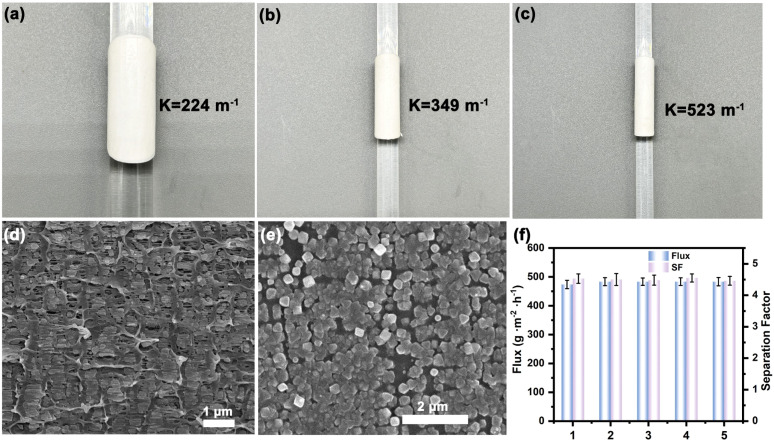
(a–c) Photographs of the UiO-66/PP-(96) membrane after bending with glass rods of varying diameters, *K* is the curvature of bending; (d) cross-section SEM image of the UiO-66/PP-(96) membrane; (e) top-view SEM image of the UiO-66/PP-(96) membrane; (f) total flux and *n*-Hex/22DMB separation factor of the UiO-66/PP-(96) membrane after bending 5 times.

In addition, after immersion in a mixture of *n*-Hex and 22DMB for two weeks, the separation performance of the UiO-66/PP-(96) membrane was retained at least 99% compared to the freshly prepared membrane (Fig. S25†). The observations suggest that the membrane structure remained intact throughout the test time, without evident leaks or blockages, showing high durability. Mechanical testing further verified the membrane robustness. The UiO-66/PP-(96) membrane was bent along glass rods with different diameters 5 times, revealing a maximum curvature tolerance of 523 m^−1^ (Fig. 5a–c). Post-bending characterization showed no cracking, delamination, or structural damage in SEM analysis (Fig. 5e and f), while retaining consistent separation performance (flux = 483.5 g m^−2^ h^−1^) with unchanged *n*-Hex/22DMB selectivity. These results show the high mechanical flexibility and stability of the mixed-matrix membrane, attributed to the flexible PP, even as a minor composition of 27.1 wt%, as well as strong binding of UiO-66 NPs with the PP substrate. The preferential permeation of *n*-Hex is governed by both kinetic and thermodynamic driving forces: lower structural constraints at the pore mouths favor the diffusion of *n*-Hex over 22DMB and enhanced thermodynamic affinity for *n*-Hex in UiO-66 cavities. These findings collectively establish UiO-66/PP membranes as a promising solution for challenging isomer separation applications.

## Conclusions

In summary, we present a facile strategy for fabricating defect-free UiO-66 mixed-matrix membranes through *in situ* confined assembly, where precursors diffused from opposite sides of PP substrates enable controlled crystal growth and dense packing of UiO-66 within the macropores under ambient conditions. This protocol effectively inhibits the abrupt nucleation and rapid agglomeration of UiO-66 in the mother solution, allowing precise formation of well-packed MOF membranes within the substrate. The resulting UiO-66/PP-(96) membrane with an ultrahigh 72.9 wt% MOF loading demonstrates exceptional separation performance, achieving a flux of 473.5 g m^−2^ h^−1^ with a separation factor of 4.55 for *n*-Hex/22DMB while retaining long-term operational stability. The membrane also shows excellent performance for other hexane isomer pairs (*n*-Hex/23DMB and 2MP/22DMB), highlighting its potential for high-RON gasoline enrichment and recycling of linear/mono-branched isomers to isomerization reactors. This fabrication strategy sheds light on resolving the long-standing problems of achieving high filler loading, agglomeration and interfacial defects in MOF-based mixed-matrix membranes for industrial applications.

## Experimental

### Experiment materials

The starting materials were acquired from commercial sources and used directly without any further purification. The detailed procedure is described in the ESI†

### Preparation of UiO-66

UiO-66 was synthesized at room temperature with reference to the literature and minor adjustments. The synthesis process primarily involved the formation of a metal cluster precursor at elevated temperatures, which was then coordinated to the ligand. The detailed experimental procedure is outlined as follows: (1) 840 mg zirconium propoxide solution (70 wt% in *n*-propanol) was dissolved in a mixture solvent of 30 mL DMF and 16 mL acetic acid, sonicated for 10 min. The transparent solution was heated at 130 °C for 3 h, during which the solution color transitioned from colorless to yellow, indicating metal cluster formation. The solution was then cooled to room temperature for subsequent reaction. (2) 420 mg of 1,4-benzenedicarboxylate was added to the cooled metal cluster solution. The mixture was ultrasonicated for 20 min and allowed to react at room temperature for 24 h to complete the coordination process. The white product was centrifuged at 13 000 rpm for 15 min, followed by three washing cycles with DMF and methanol, and finally dried at 80 °C for 24 h.

### Preparation of UiO-66/PP membranes

The metal cluster precursor was obtained following the protocol of step 1. The ligand solution was prepared as follows: 1,4-benzenedicarboxylic acid (420 mg) was charged into a screw-topped glass bottle, and then 46 mL DMF was added. A transparent solution was obtained after ultrasonication for 20 min. Polypropylene (PP) membranes were trimmed into circular discs with a diameter of 25 mm and soaked in DMF for 10 min to ensure complete wetting. Subsequently, these membranes were then fixed at the center of a laboratory-made anti-diffusion device (Fig. S26†). The metal cluster precursor and ligand solutions were added on opposite sides of the substrate for reactions of 24, 72, and 96 h, respectively. The resultant membranes were immersed in a methanol solution for 12 h with replacement of fresh methanol solution every 3 h to remove residual reactants. Final drying at 60 °C for 24 h yielded the UiO-66/PP membranes, denoted as UiO-66/PP-(24), UiO-66/PP-(72), and UiO-66/PP-(96) according to the reaction time.

### Hexane isomer pervaporation test

The pervaporation device consists of a membrane-based component assembly, a product collection module, and an integrated vacuum system. UiO-66/PP membranes with an effective area of 5 cm^2^ were fixed in a home-made membrane chamber and sealed with a perflurane O-ring and then connected to the pervaporation system. Generally, a peristaltic pump is employed to deliver the feed liquid of the mixed hexane isomers (*n*-Hex/22DMB, *n*-Hex/23DMB, 2MP/22DMB) with equal mass ratio (1 : 1) to the front side of the membrane, and an oil pump maintains a vacuum on the permeate side, upon reaching steady-state in pervaporation. The permeant was collected in a liquid nitrogen cold trap. After weighing the collected liquids with an electronic balance, the composition of the permeant was analyzed by gas chromatography. Moreover, the pervaporation experiment was conducted in triplicate for each membrane to enhance the reliability of the results. The permeation flux was determined utilizing the following equation:1
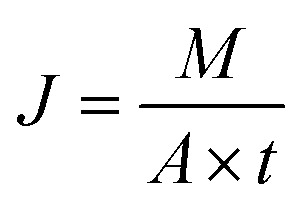
where *M* is the mass of the permeant (g), *A* is the effective membrane area (m^2^) and *t* is the time (s) corresponding to the permeation experiment. The permeance of each component was obtained *via*:2
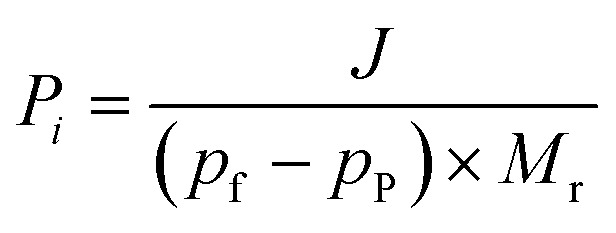
where *p*_f_ (kPa) and *p*_p_ (kPa) denote the vapor pressure of the target component on the feed and permeate sides, respectively. *p*_f_ was determined by using Antoine coefficients, while *p*_p_ was assumed negligible under vacuum conditions of pervaporation. *M*_r_ (g mol^−1^) represents the molar weight of the component. The permeability is obtained using the following formula.3
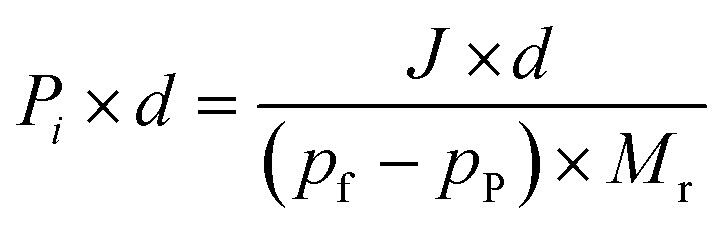
where *d* is the thickness of the membrane. The separation factor of the membrane quantifies the degree of separation achieved between the two substances by the permeable membrane. A higher value signifies a more effective separation. This is described using the following equation:4
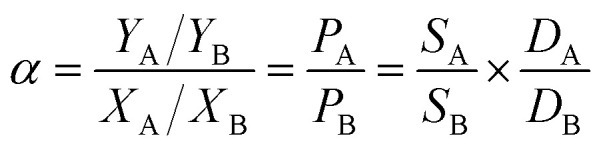
where *Y*_A_ and *Y*_B_ refer to the mass fractions of substances A and B in the permeant side, *X*_A_ and *X*_B_ represent the mass fractions of substances A and B in the feed solution, and *α* denotes the separation factor. *P*_A_, *P*_B_, *S*_A_, *S*_B_, *D*_A_ and *D*_B_ are the permeability, solubility and diffusivity of components A and B, respectively. *S*_A_ and *S*_B_ can be calculated from fitting the adsorption data with the Langmuir–Freundlich model (eqn (5)).5
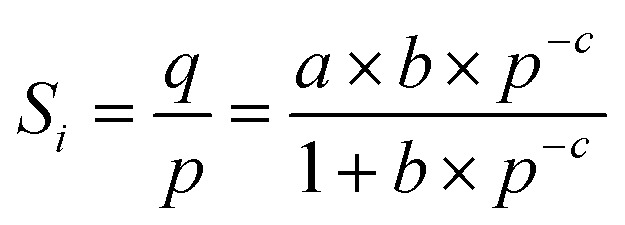


*D*_A_ and *D*_B_ can be derived from eqn (6):

6 *P* = *S* × *D*

In addition, the pervaporation separation index is also used to reflect the performance of the membrane, and its calculation formula is as follows:

7 PSI = *J* × (*α* − 1)

## Author contributions

Y. L., Q. G. and M. X. conceived and designed this work. P. Z. conducted the synthesis and characterization of the materials and wrote this paper. J. Y. and W. L. helped with the characterization of the materials. Z. C., W. S. and Y. C. helped with the data analysis and discussion. All authors reviewed and edited the manuscript.

## Conflicts of interest

There is no conflict of interest to declare.

## Supplementary Material

SC-OLF-D5SC04212G-s001

SC-OLF-D5SC04212G-s002

## Data Availability

The data supporting this article have been included as part of the ESI.†
