# Supplementary material for: Confined growth of UiO-66 into ultrahigh-loading membranes for efficient hexane isomer separation
Source: Chem Sci. 2025 Jul 19;16(33):15206–15. doi: 10.1039/d5sc04212g (PMC12287939; doi:10.1039/d5sc04212g)
Supplement: SC-016-D5SC04212G-s002 [file SC-016-D5SC04212G-s002.pdf]

Supplementary Information (SI) for Chemical Science.
This journal is © The Royal Society of Chemistry 2025

Supporting Information

Confined Growth of UiO-66 into Ultrahigh-loading Membrane for Efficient Hexane Isomer Separation

Pan-Pan Zhang,[a] Jing-Ran Yu,[a] Jia-Yu Ding,[b] Wei-Hai Lin,[a] Zhen Chen,[a] Wei Shao,[a] Shu-Chang Wang [a], Yi-Le Chen,[a] Yi Li,*[a] Qi-Han Gong,*[b] Ming Xue,*[a] Xiao-Ming Chen[a]

[a]School of Chemical Engineering and Technology, Southern Marine Science and Engineering Guangdong Laboratory (Zhuhai), MOE Key Laboratory of Bioinorganic and Synthetic Chemistry, GBRCE for Functional Molecular Engineering, School of Chemistry, IGCME, Zhuhai Key Laboratory of Optoelectronic Functional Materials and Membrane Technology, Sun Yat-sen University, Guangzhou 510275, China.

[b]Fundamental Science & Advanced Technology Lab, PetroChina Petrochemical Research Institute, Beijing, 102200, P. R. China.

**Corresponding Author: xueming5@mail.sysu.edu.cn; liyi266@mail.sysu.edu.cn; gongqihan@petrochina.com.cn*

Experimental Section

1. Chemicals

Zirconium(IV) *n*-propoxide, 70% w/w in *n*-propanol, packaged under Argon in resealable bottles ($C_{12}H_{28}O_4Zr$, 70 wt%, Alfa), 1,4-benzenedicarboxylic acid ($C_8H_6O_4$, 99% purity, Aladdin), 25 μm porous polypropylene (PP) membrane (Celgard 2500, Jinghong New Energy Technology Co., Ltd), acetic acid ($C_2H_4O_2$, 99% purity, Aladdin), *N*,*N*-dimethylformamide (C_3H_7NO, 99.5% purity, Macklin), methanol (CH_4O, 99% purity, Xilong Scientific Co., Ltd), *n*-hexane (C_6H_{14}, 99% purity, Aladdin), 2-methylpentane (C_6H_{14}, 99% purity, Aladdin), 2,2-dimethylbutane (C_6H_{14}, 99% purity, Aladdin), 2,3-dimethylbutane (C_6H_{14}, 99% purity, Aladdin).

2. Characterization

The SEM images of both UiO-66 nanocrystals and membranes were observed from the ultrahigh resolution thermal field emission scanning electron microscope (Thermoscientific Apreo 2S HiVac), equipped with an energy spectrum diffractometer (Oxford Ultim Max65). The surface morphology and roughness were achieved through AFM (atomic force microscopy) instruments (Dimension Icon Bruker). Wide angle X-ray was used to characterize the structure of UiO-66, and the XRD patterns were obtained from Rigaku Ultima IV with a Cu-Kα radiation of $\lambda = 0.154$ nm (40 kV, 40 mA), and a scan rate of 2° min^{-1}. The nitrogen gas adsorption-desorption experiment was carried to analyze the pore structure of UiO-66 at 77 K on micromeritics instrument 2460. Single-component vapor adsorption isotherms of *n*-Hex, 2MP, 22DMB and 23DMB on UiO-66 were performed on Belsorp Max II. Before the experiment, the material was activated at 393 K for 8 h under vacuum. Fourier transform infrared (FTIR) spectra were recorded on a Nicolet iS50 spectrometer. The actual loadings of the UiO-66/PP membrane were measured on thermogravimetric analyzer Netzsch TG209F1 Libra, treating from 303 K to 1273 K with a heating rate of 10 °C min^{-1} under a continuous air atmosphere. Elemental analysis was performed by an inductively coupled plasma atomic emission spectrometer (ICP-AES) on OPtima8300. X-ray photoelectron spectroscopy was performed on SHIMADZU AXIS SUPRA$^+$.

3. **Preparation of UiO-66/PP_{MF}-(96) membrane.**

The metal cluster precursor was synthesized following Step 1 of the UiO-66. A ligand solution was prepared by dissolving 1,4-benzenedicarboxylic acid (H_2BDC, 420 mg, 99%) in *N*,*N*-dimethylformamide (DMF, 46 mL) under sonication for 20 min. A polypropylene microfiltration membrane (PP_{MF} membrane, 25 mm diameter) was pre-wetted by soaking in DMF (20 mL) for 10 min and subsequently mounted in a custom-built diffusion cell (Fig. S25). The metal cluster precursor solution and ligand solution were introduced on opposite sides of the membrane and allowed to react for 96 h at ambient temperature. The resulting composite membrane was immersed in methanol for 24 h to remove residual reactants, followed by drying at 60 °C for 24 h under vacuum. Samples were designated as UiO-66/PP_{MF}-(96).

4. Preparation of ZIF-8/PP-(96) membrane.

To create ZIF-8/PP-(96) membrane, the contra-diffusion method was used. Two solutions containing 586 mg Zn $(NO_3)_2\cdot 6H_2O$ in 40 mL distilled water and 1.3 g 2-methylimidazole in 40 mL distilled water were prepared. The remaining steps are consistent with the preparation of UiO-66/PP-(96) membrane.

5. Molecular Dynamics (MD) Simulations

Molecular Dynamics (MD) simulations of the directed diffusion of these four different isomers of hexane in UiO-66 metal organic framework material were carried out using the Gromacs program suite, with the

hybridized force field of TraPPE-UA force field and UFF4MOFII force field. The molecules of these four organic molecules were simulated using TraPPE-UA parameters. The UiO-66 slab models were parameterized using UFF4MOFII forcefield and REPEAT atomic charge. All these topology files of these molecules and crystals were generated directly using AuToFF web server. The initial simulation boxes of (1 1 0) lattice plane which has two vacuum layers of 4 nm thickness and a layer of graphene was also inserted to separation them because periodic boundary conditions were applied in all directions. There were 600 hexane in one side of the layers at the initial state. The structures were first energy-minimized and then a MD simulation for a total simulation time of 100 ns were performed at constant NVT ensemble, and the trajectory was saved every 50 ps. In order to accelerates the diffusion process, the temperature was maintained at 400 K using the velocity-rescale thermostat with a relaxation constant of 1 ps and the electrostatic interactions and van der Waals forces were treated using the Particle-mesh Ewald (PME) method with a cut-off distance of 15 Å. In order to compare the directed diffusion quantitatively, the mean square displacement of these molecules along the z axis (i.e., the direction perpendicular to the surface of the MOF material) over time were calculated using the Gromacs tool-suites. The initial and final snapshots of these diffusion simulation process were rendered using the Visual Molecular Dynamic program (VMD).

Supporting Figures and Tables

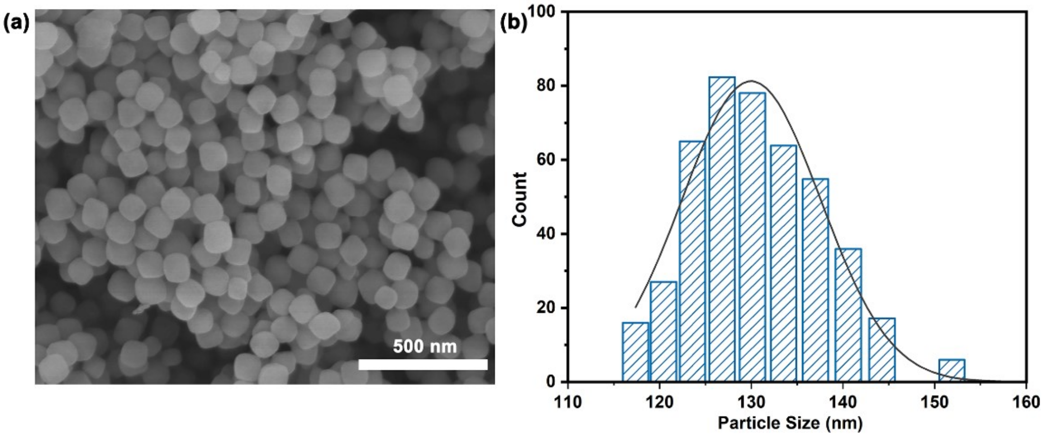

Fig. S1 Scanning Electron Microscopy (SEM) image of nanosized UiO-66 synthesized at ambient temperature (a); Statistical particle size distribution (>100 particles) (b).

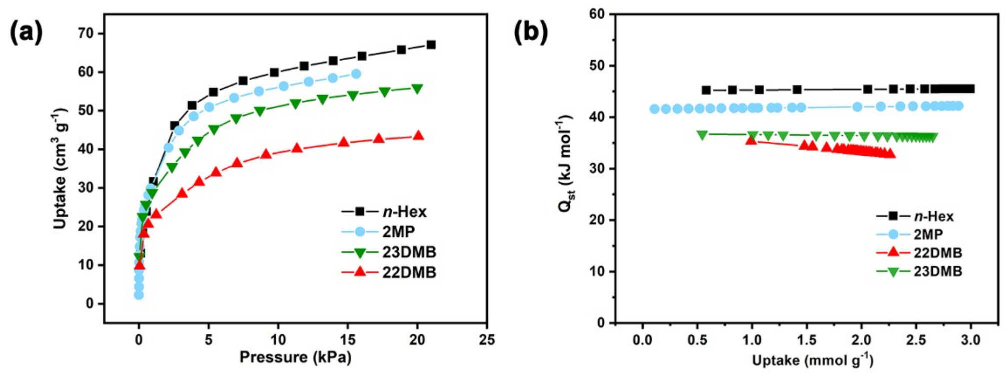

Fig. S2 Adsorption isotherms of *n*-Hex, 2MP, 23DMB and 2,2DMB on UiO-66 at 323 K (a); Isosteric heats of *n*-Hex, 2MP, 23DMB and 2,2DMB as a function of its uptake (b).

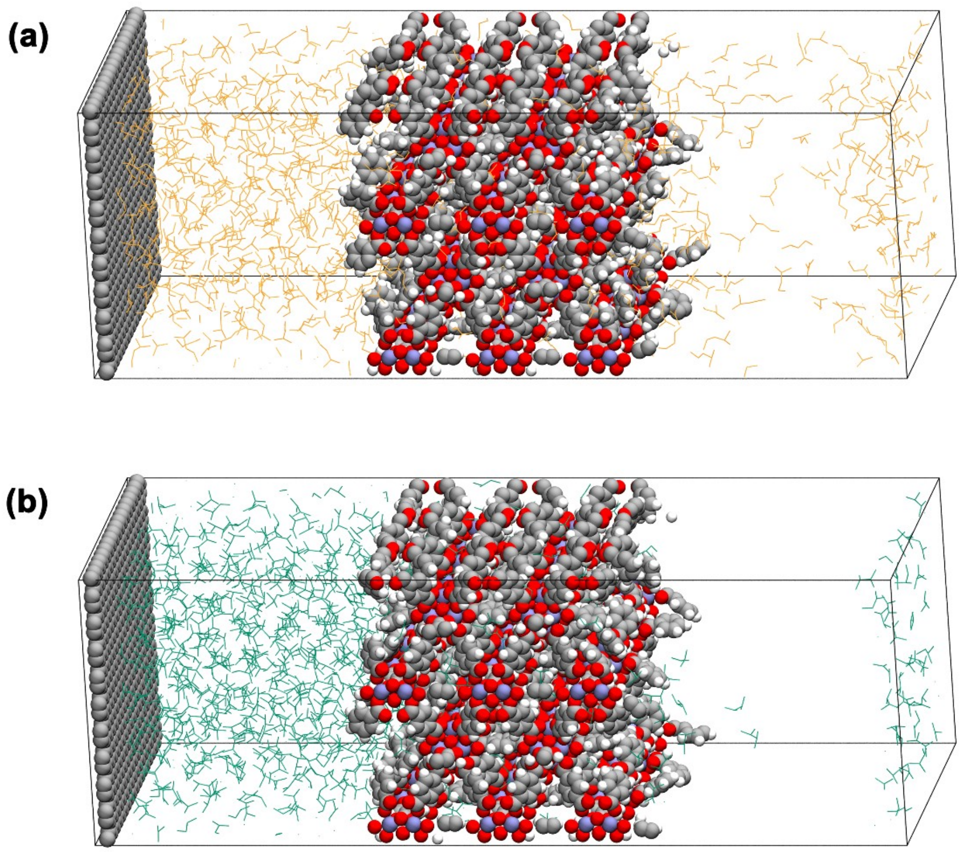

Fig. S3 The diffusion behaviors of 2-MP (orange) (a) and 23DMB (blue) (b) on UiO-66 obtained by MD simulation

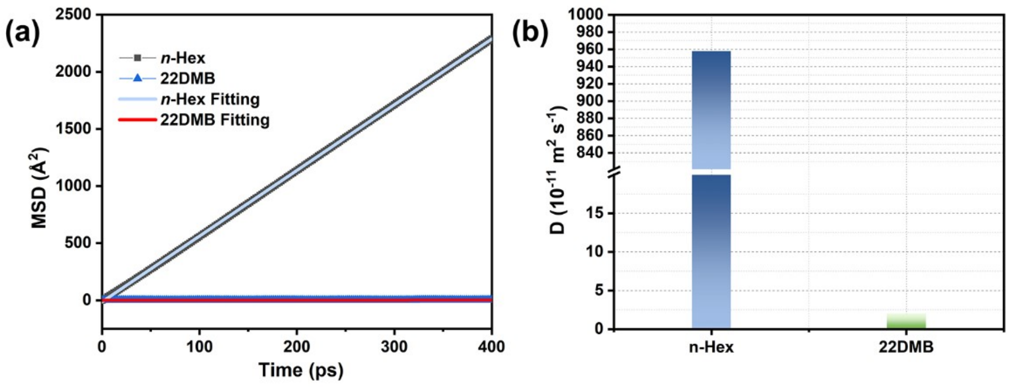

Fig. S4 MSD plots (a) and self-diffusion coefficient (b) of *n*-Hex and 22DMB in UiO-66 after change the inital configuration.

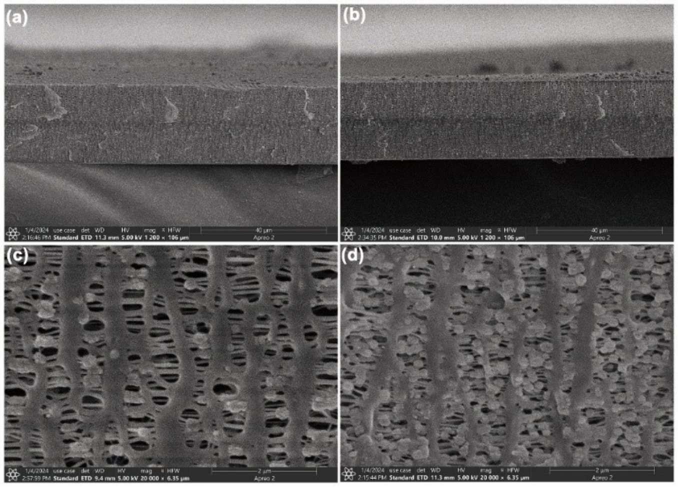

Fig. S5 Cross-section SEM images for membranes of UiO-66/PP-(24) (a); UiO-66/PP-(72) (b); (c) and (d) are corresponding enlarged views of (a) and (b), respectively.

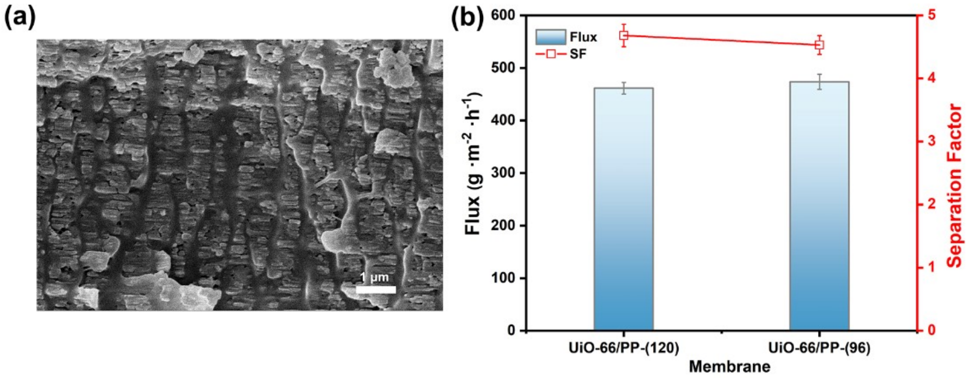

Fig. S6 Cross-section SEM image of UiO-66/PP-(120) (a); Total fluxes and *n*-Hex/22DMB separation factors measured on UiO-66/PP-(120) and UiO-66/PP-(96) membrane (b).

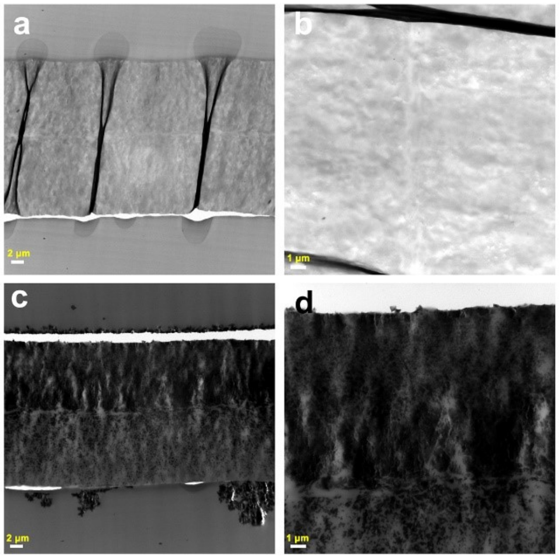

Fig. S7 TEM images for PP (a, b) and UiO-66/PP-(96) (c, d).

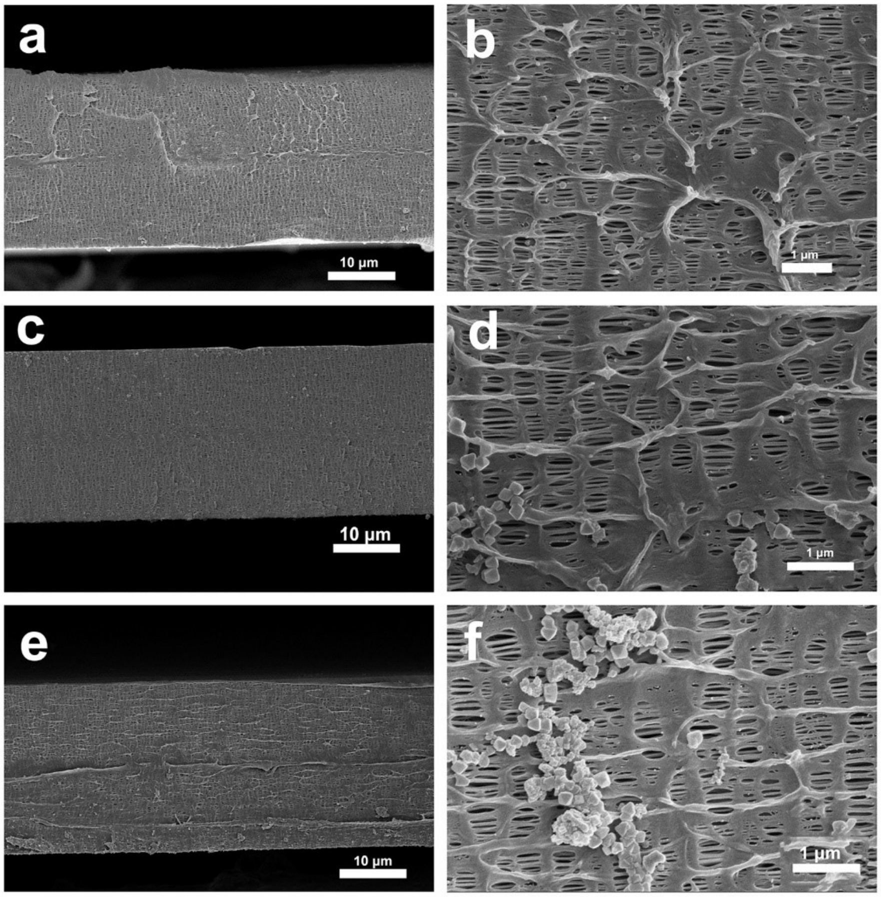

Fig. S8 SEM images for UiO-66/PP-(24_{dc}) (a, b), UiO-66/PP-(72_{dc}) (c, d), and UiO-66/PP-(96_{dc}) (e, f).

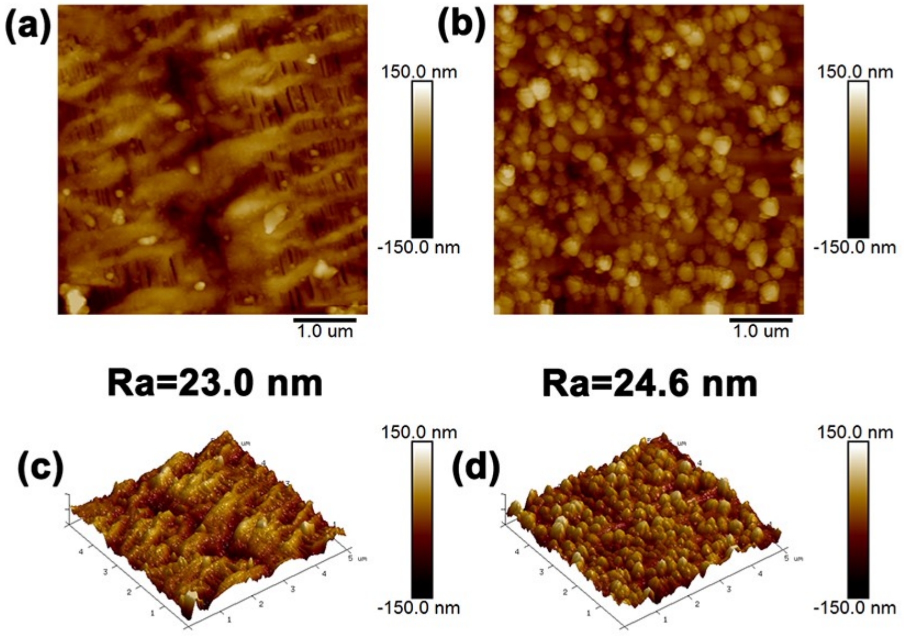

Fig. S9 Top surface AFM images of UiO-66/PP-(24) (a) and UiO-66/PP-(72) (b); (c) and (d), are corresponding 3D height images of (a) and (b), respectively.

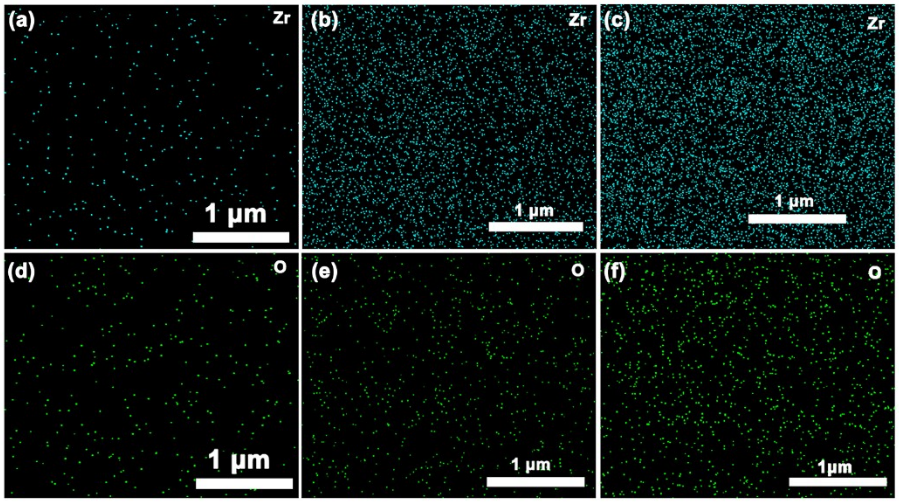

Fig. S10 Zr and O Elemental mapping of UiO-66/PP-(24) (a, d); UiO-66/PP-(72) (b, e); UiO-66/PP-(96) (c, f).

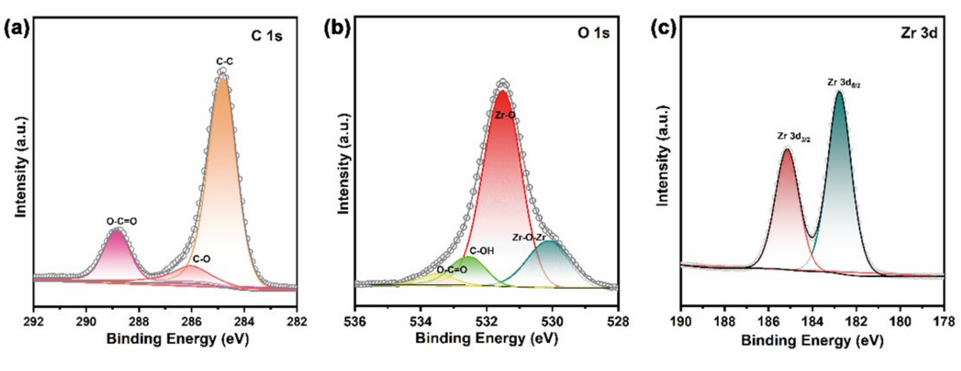

Fig. S11 High resolution XPS spectra of UiO-66 for C 1s (a); O 1s (b); Zr 3d (c).

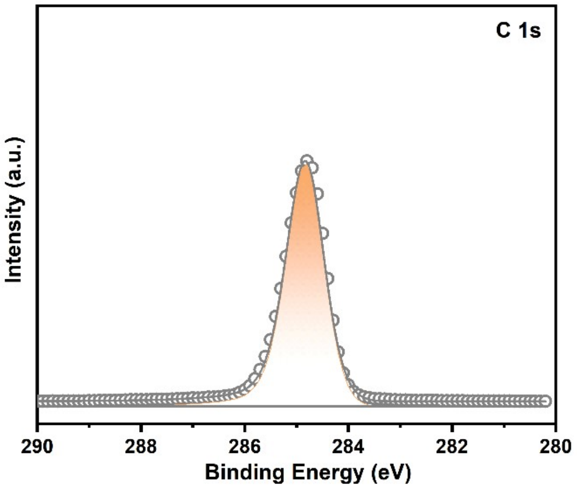

Fig. S12 High resolution XPS spectra of PP membrane for C 1s.

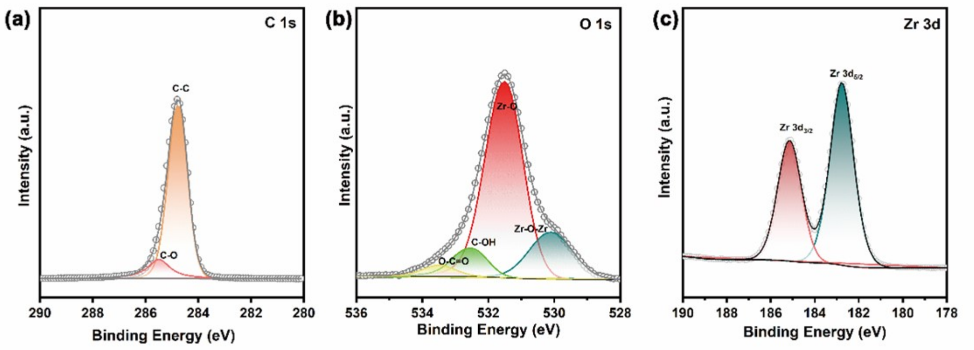

Fig. S13 High resolution XPS spectra of UiO-66/PP-(24) for C 1s (a); O 1s (b); Zr 3d (c).

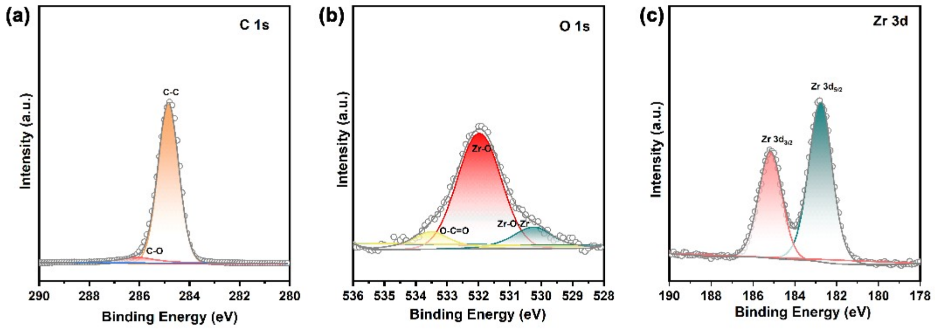

Fig. S14 High resolution XPS spectra of UiO-66/PP-(72) for C 1s (a); O 1s (b); Zr 3d (c).

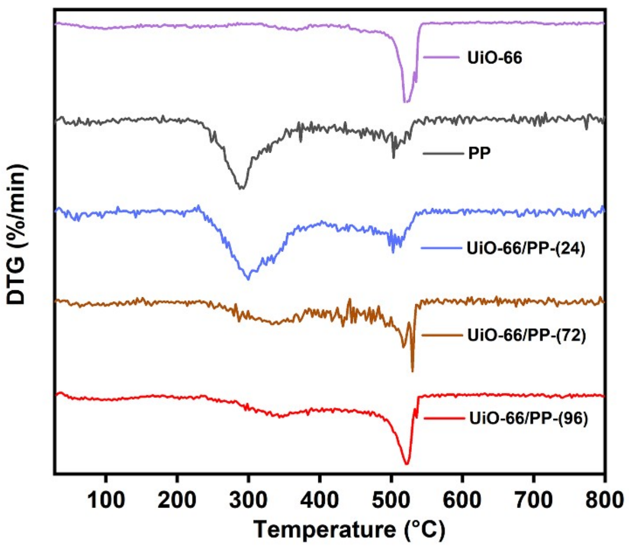

Fig. S15 Derivative TG (DTG) curves of UiO-66, PP and UiO-66/PP membrane.

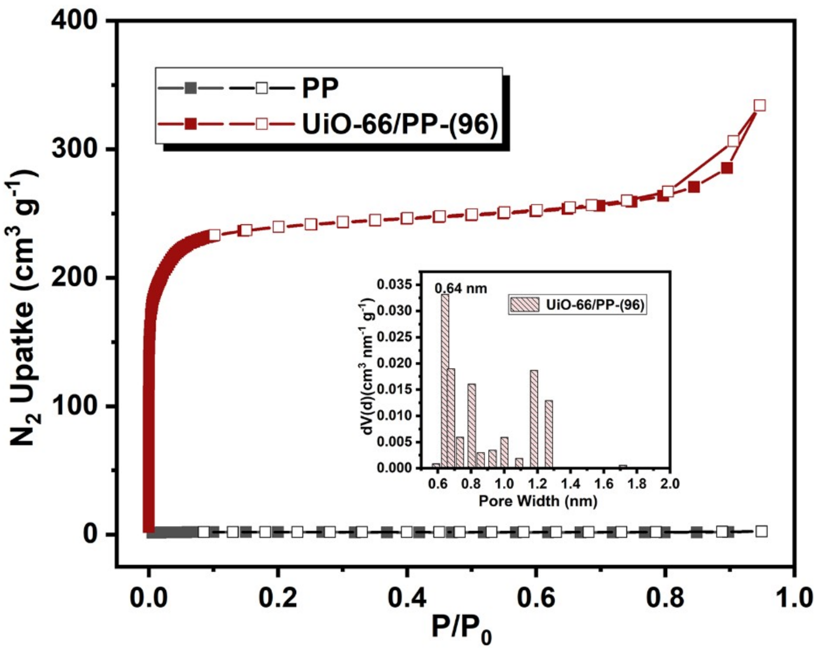

Fig. S16 N_2 adsorption-desorption isotherm measured at 77 K for UiO-66/PP-(96) and pristine PP membrane, inset shows the pore size distribution of UiO-66/PP-(96) membrane.

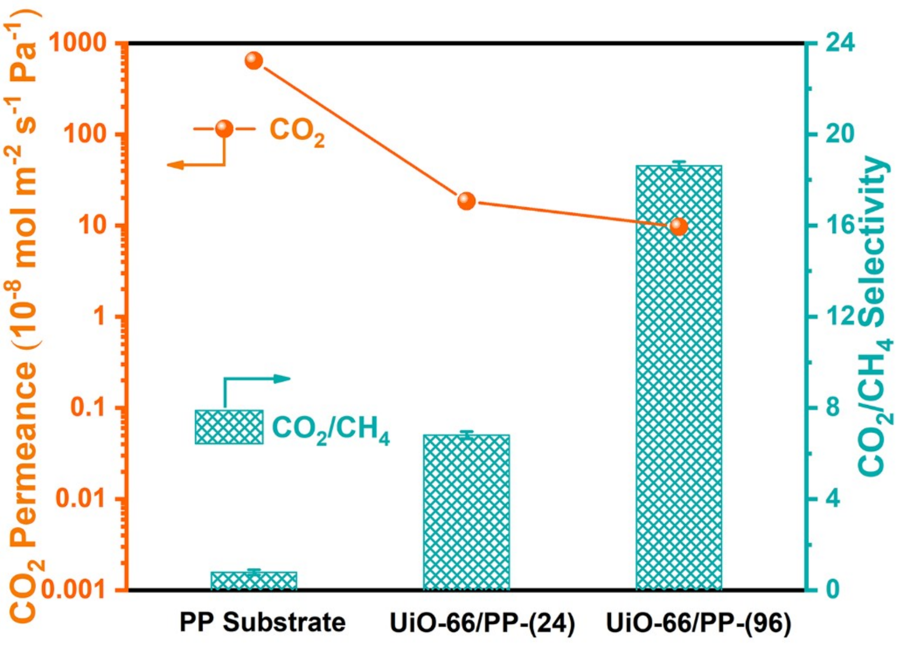

Fig. S17 CO_2 permeance and CO_2/CO_4 selectivity for single gas through the neat membrane of PP and membranes of UiO-66/PP-(24) and UiO-66/PP-(96).

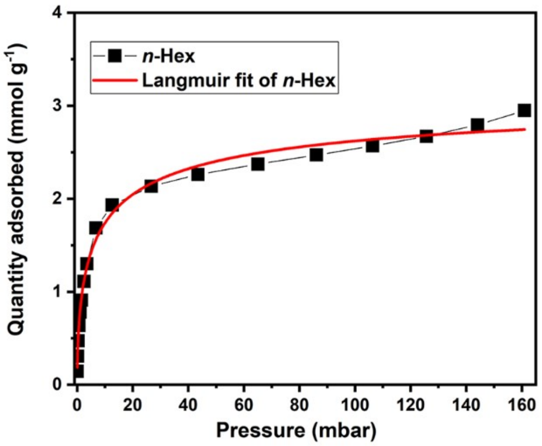

Model	Langmuir Freundlich
Equation	y=(a*b*x^(1-c))/(1+b*x^(1-c))
a	3.23729 ± 0.17715
b	0.31683 ± 0.02462
c	0.43587 ± 0.04384
Adj. R-Square	0.99217

Fig. S18 Adsorption isotherms of *n*-Hex on the UiO-66/PP-(96) membrane.

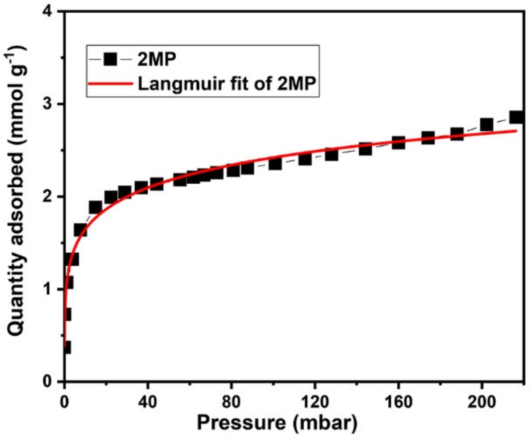

Model	Langmuir Freundlich
Equation	y=(a*b*x^(1-c))/(1+b*x^(1-c))
a	6.38864 ± 1.41454
b	0.21037 ± 0.05745
c	0.74859 ± 0.02731
Adj. R-Square	0.98983

Fig. S19 Adsorption isotherms of 2MP on the UiO-66/PP-(96) membrane.

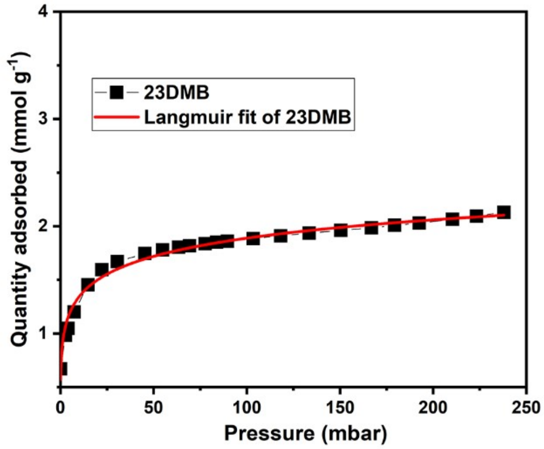

Model	Langmuir Freundlich
Equation	y=(a*b*x^(1-c))/(1+b*x^(1-c))
a	3.90646 ± 0.77922
b	0.29293 ± 0.06996
c	0.74769 ± 0.03418
Adj. R-Square	0.98453

Fig. S20 Adsorption isotherms of 23DMB on the UiO-66/PP-(96) membrane.

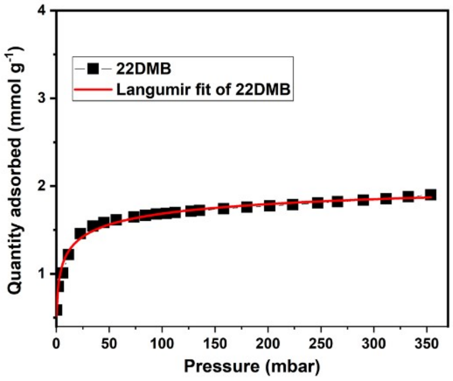

Model	Langmuir Freundlich
Equation	y=(a*b*x^(1-c))/(1+b*x^(1-c))
a	2.25091 ± 0.08732
b	0.47622 ± 0.02565
c	0.60167 ± 0.02961
Adj. R-Square	0.9891

Fig. S21 Adsorption isotherms of 22DMB on the UiO-66/PP-(96) membrane.

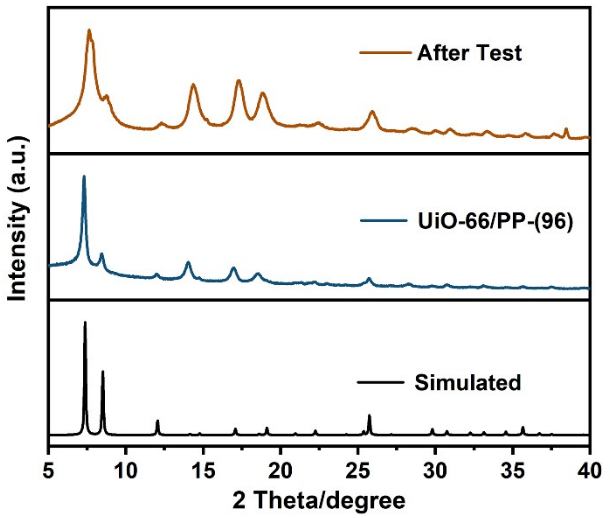

Fig. S22 XRD patterns of UiO-66/PP-(96) membrane after 48 h pervaporation test.

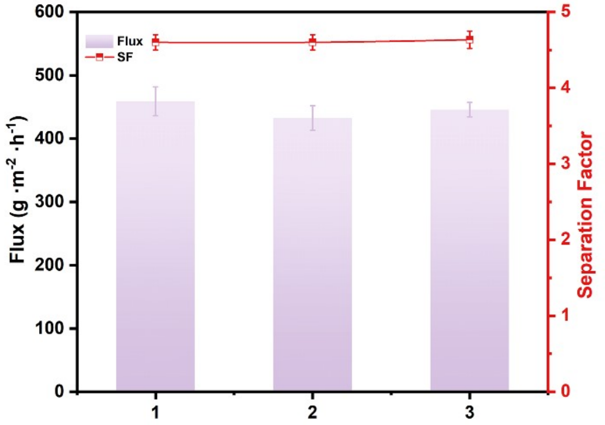

Fig. S23 Flux and *n*-Hex/22DMB separation factor for UiO-66/PP-(96) membrane of 5vol% toluene and 5vol% m-Xylene added.

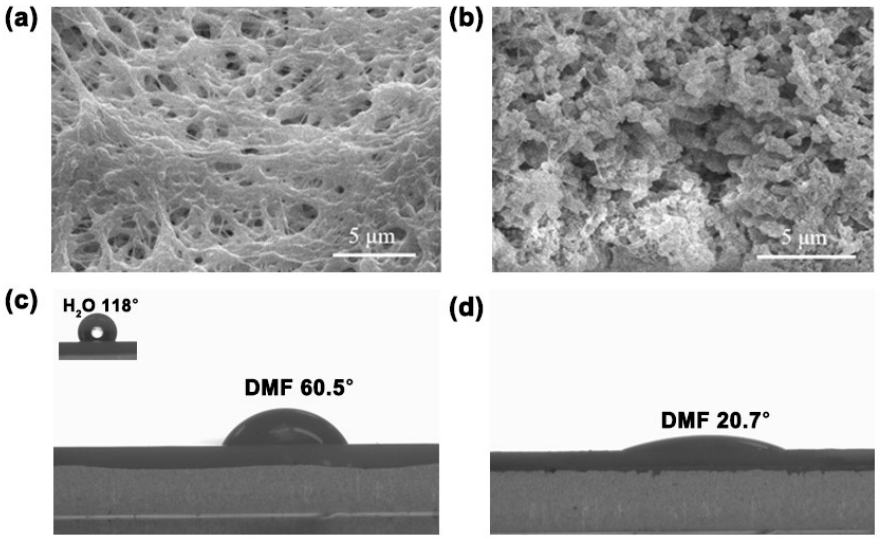

Fig. S24. Cross-sectional SEM images for pristine microfiltration PP (a) and UiO-66/PP_{MF}-(96). Contact angle measurements for PP with DMF (inset: water contact angle on PP) (c) and microfiltration PP with DMF (d).

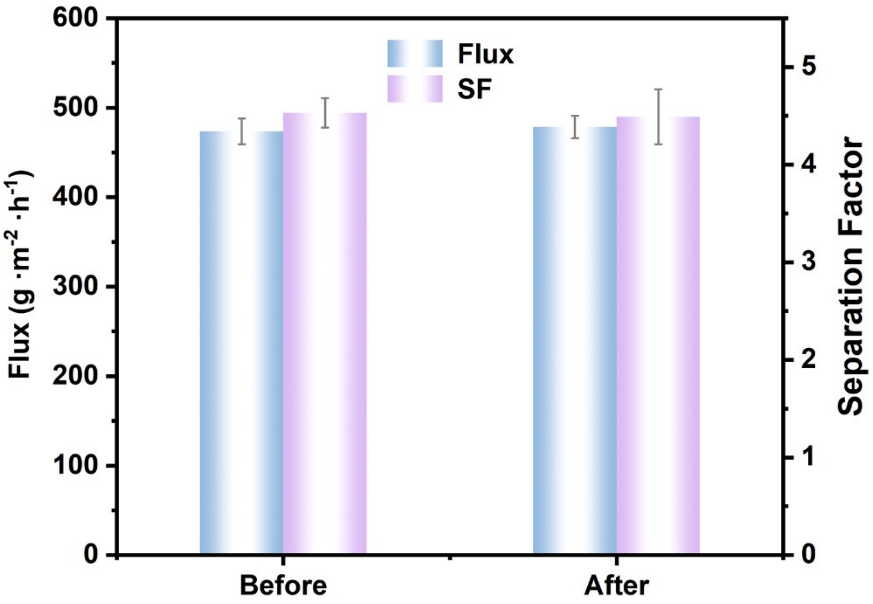

Fig. S25 Pervaporation separation of equal mass fraction *n*-Hex/22DMB for UiO-66/PP-(96) membrane before and after two week-long swelling tests

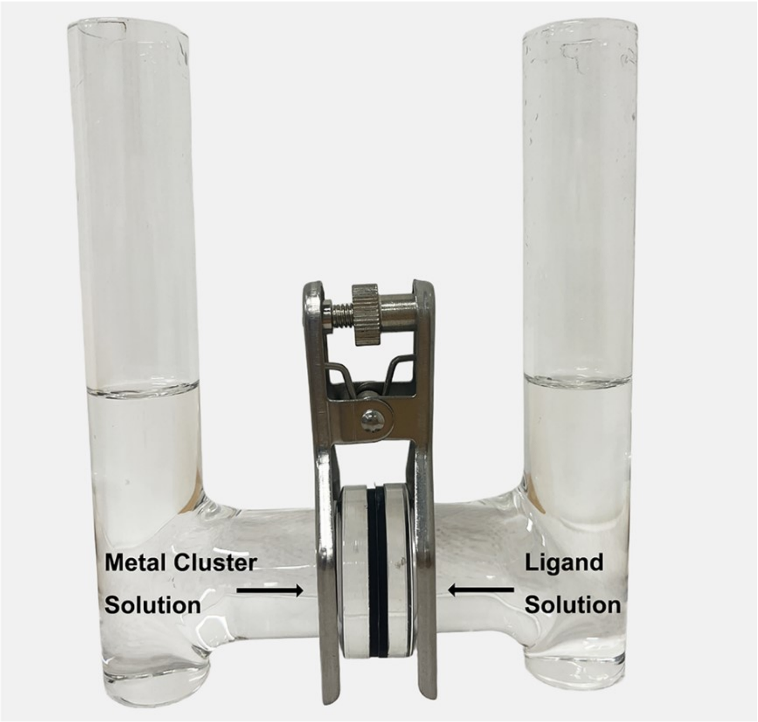

Fig. S26 The laboratory-made anti-diffusion device.

Table S1. The introduction overlooks recent advances in alkane-selective MOFs.

MOF	Surface Area (m^2 g^{-1})	Hexane Uptake (mg g^{-1})	Ref.
NU-80-LP	670	220	S1
HIAM-302	388	96	S2
HIAM-318	599	115	S3
NU-2200	355	89	S4
NU-2005	380	57	S5
KMF-1	943	154	S6
Cu-WO_4-TPA	146	70	S7
Ni-Asp	217	177	S8
Zn(BDC)(Dabco)$_{0.5}$	1450	14.6	S9
$Fe_2(BDP)_3$	1230	133	S10
Zr-abtc	1318	80	S11
ZIF-8	1836	236	S12
MIL-140B	460	42	S13
MIL-160(Al)	911	117	S14
$Zn_4O(NTB)_2$	622	215	S15
Al-bttotb	572	148	S16
UiO-66	1340	340.5	***This work***

Table S2. ICP-AES and TGA analysis for the UiO-66/PP membranes.

Membrane	ICP	TGA
UiO-66/PP-(24)	20.8%	22.5%
UiO-66/PP-(72)	48.3%	52.3%
UiO-66/PP-(96)	70.4%	72.9%
UiO-66/PP-(120)	72.3%	73.1%

Table S3. Pervaporation for single-component through UiO-66/PP-(96) membrane.

Isomer	Performance	
	Flux ($g \cdot m^{-2} \cdot h^{-1}$)	Permeance ($mol\ m^{-2}\ s^{-1}\ Pa^{-1}$)
n-Hex	401.9 ± 7.6	6.4×10^{-8}
2MP	371.3 ± 7.6	5.9×10^{-8}
23DMB	166.0 ± 27.3	2.6×10^{-8}
22DMB	91.7 ± 13.1	1.5×10^{-8}

Table S4. The solubility (S) and diffusion (D) coefficients a for UiO-66/PP-(96) membrane derived from the adsorption isotherm and single-component permeation data.

Isomer	Solubility coefficient ($mmol\ g^{-1}\ mbar^{-1}$)	Diffusion coefficient ($m^2\ s^{-1}$)
n-Hex	1.38×10^{-2}	9.69×10^{-12}
2MP	1.31×10^{-2}	9.45×10^{-12}
23DMB	7.68×10^{-3}	7.21×10^{-12}
22DMB	4.40×10^{-3}	6.95×10^{-12}

Table S5. The solubility selectivity and diffusion selectivity for UiO-66/PP-(96) membrane.

Selectivity	*n*-Hex/22DMB	*n*-Hex/23DMB	2MP/23DMB
Solubility	3.14	1.80	2.98
Diffusion	1.39	1.34	1.36

Table S6. The pervaporation separation index of the membranes.

Membrane	PSI ($g \cdot m^{-2} \cdot h^{-1}$)
PP (n-Hex/22DMB)	250.7
UiO-66/PP-(24) (n-Hex/22DMB)	274.1
UiO-66/PP-(72) (n-Hex/22DMB)	838.8

UiO-66/PP-(96) (n-Hex/22DMB)	1671.5
UiO-66/PP-(96) (n-Hex/23DMB)	1128.8
UiO-66/PP-(96) (2MP/22DMB)	1315.5

Table S7. Flux and Separation Factor for UiO-66/PP through conventional methods of dip-coating.

**Membrane**	**Flux** ($g \cdot m^{-2} \cdot h^{-1}$)	**Separation Factor**
UiO-66/PP-(24_{dc})	1099	1.22
UiO-66/PP-(72_{dc})	1089	1.23
UiO-66/PP-(96_{dc})	1097	1.22

Table S8. Separation performance of the UiO-66/PP-(96) membrane in this work compared with that of the reported membranes.

**Membrane**	**Performance**		**Ref.**
	**Flux** ($g\ m^{-2}\ h^{-1}$)	**Separation Factor**	
Zeolite Beta	5.01	1.81 (*n*-Hex/22DMB)	S17
Zeolite Beta	5.01	1.64 (*n*-Hex/23DMB)	S17
Silicalite-1-M2	56.34	98 (*n*-Hex/2MP)	S18
Silicalite-1-LM1	20.80	36 (*n*-Hex/2MP)	S18
MFI	6.87	50 (*n*-Hex/23DMB)	S19
H-ZSM-5	25.59	9.32 (*n*-Hex/22DMB)	S20
NaY-Zeolite	118.59	1.90 (*n*-Hex/DMB)	S21
PTMSP-MFI1	0.18	3.60 (*n*-Hex/DMB)	S22
PTMSP-MFI2	0.19	3.98 (*n*-Hex/DMB)	S22
PTMSP-MFI3	0.16	3.24 (*n*-Hex/DMB)	S22
Al-bttotb	40	3.85 (*n*-Hex/22DMB)	S23
Al-bttotb	35	2.01 (3MP/22DMB)	S23
HKUST-1	1061	51.6 (*n*-Hex/23DMB)	S24
UiO-66/PIM-1	1745	3.14 (*n*-Hex/22DMB)	S25
UiO-66/PIM-1	1703	2.29 (2MP/22DMB)	S25

UiO-66/PP-(96)	**473.52**	**4.53 (*n*-Hex/22DMB)**	***This Work***
UiO-66/PP-(96)	**462.63**	**3.44 (*n*-Hex/23DMB)**	***This Work***
UiO-66/PP-(96)	**424.62**	**4.09 (2MP/22DMB)**	***This Work***

Table S9 Flux and Separation Factor measured on membrane using different MOF and polymer.

**Membrane**	**Flux** ($g \cdot m^{-2} \cdot h^{-1}$)	**Separation Factor**
ZIF-8/PP-(96)	982	1.8
UiO-66/PP_{MF}-(96)	1087	1.2

Supplementary References:

[S1] Z. J. Jiang, Y. Wang, D. Luo, R.-J. Wei, W. Lu and D. Li, Dehydration-Induced Cluster Consolidation in a Metal-Organic Framework for Sieving Hexane Isomers, *Angew. Chem. Int. Ed.*, 2024, **63**, e202403209.
[S2] L. Yu, S. Ullah, K. Zhou, Q. Xia, H. Wang, S. Tu, J. Huang, H.-L. Xia, X.-Y. Liu, T. Thonhauser and J. Li, A Microporous Metal-Organic Framework Incorporating Both Primary and Secondary Building Units for Splitting Alkane Isomers, *J. Am. Chem. Soc.*, 2022, **144**, 3766-3770.
[S3] X. Zhou, L. L. Ma, L. Yu, K. Zhou, K. Xiong, Y. Gai, J. Li and H. Wang, Size-Exclusion Separation of Hexane Isomers by a Y-MOF Built on $\{Y(COO)_3\}_n$ Chains, *ACS Materials Lett.*, 2024, **6**, 928-932.
[S4] B. Lal, K. B. Idrees, H. Xie, C. S. Smoljan, S. Shafaie, T. Islamoglu and O. K. Farha, Pore Aperture Control Toward Size-Exclusion-Based Hydrocarbon Separations, *Angew. Chem. Int. Ed.*, 2023, **62**, e202219053.
[S5] K. B. Idrees, K. O. Kirlikovali, C. Setter, H. Xie, H. Brand, B. Lal, F. Sha, C. S. Smoljan, X. Wang, T. Islamoglu, L. K. Macreadie and O. K. Farha, Robust Carborane-Based Metal-Organic Frameworks for Hexane Separation, *J. Am. Chem. Soc.*, 2023, **145**, 23433-23441.
[S6] J. Hua, Y. Gu, Z. Li, L. Wu, H. Lian, L. Zhang, R. Hou, Y. Pan and W. Xing, Robust Aluminum-Based Metal-Organic Framework Adsorbents with Heteroatom-Functionalized Nanochannels for Hexane Isomer Separation, *Ind. Eng. Chem. Res.*, 2025, **64**, 2247-2255.
[S7] R. Chen, F. Zheng, J. Li, Y. Liu, F. Zhou, H. Sun, Q. Yang, Z. Zhang, Q. Ren and Z. Bao, Aperture Fine-Tuning in Cage-Like Metal-Organic Frameworks via Molecular Valve Strategy for Efficient Hexane Isomer Separation, *Small Struct.*, 2024, **5**, 2300302.
[S8] R. Chen, F. Zhou, B. Sheng, Z. Zhang, Q. Yang, Y. Yang, Q. Ren and Z. Bao, Robust Nickel Aspartate Framework for Shape Recognition of Hexane Isomers, *ACS Sustainable Chem. Eng.*, 2022, **10**, 11330-11337.
[S9] P. S. Bárcia, F. Zapata, J. A. C. Silva, A. E. Rodrigues and B. Chen, Kinetic Separation of Hexane Isomers by Fixed-Bed Adsorption with a Microporous Metal-Organic Framework, *J. Phys. Chem. B.*, 2007, **111**, 6101-6103.
[S10] Z. R. Herm, B. M. Wiers, J. A. Mason, J. M. van Baten, M. R. Hudson, P. Zajdel, C. M. Brown, N. Masciocchi, R. Krishna and J. R. Long, Separation of Hexane Isomers in a Metal-Organic Framework with Triangular Channels, *Science*, 2013, **340**, 960-964.
[S11] H. Wang, X. Dong, J. Lin, S. J. Teat, S. Jensen, J. Cure, E. V. Alexandrov, Q. Xia, K. Tan, Q. Wang, D. H. Olson, D. M. Proserpio, Y. J. Chabal, T. Thonhauser, J. Sun, Y. Han and J. Li, Topologically

guided tuning of Zr-MOF pore structures for highly selective separation of C_6 alkane isomers, *Nat. Commun.*, 2018, **9**, 1745.
[S12] A. Henrique, A. E. Rodrigues and J. A. C. Silva, Fixed bed dynamics of single and multicomponent adsorption of pentane and hexane isomers in ZIF-8, *Sep. Purif. Technol.*, 2020, **238**, 116419.
[S13] A. Henrique, T. Maity, H. Zhao, P. F. Brântuas, A. E. Rodrigues, F. Nouar, A. Ghoufi, G. Maurin, J. A. C. Silva and C. Serre, Hexane isomers separation on an isoreticular series of microporous Zr carboxylate metal organic frameworks, *J. Mater. Chem. A.*, 2020, **8**, 17780-17789.
[S14] P. F. Brântuas, A. Henrique, M. Wahiduzzaman, A. von Wedelstedt, T. Maity, A. E. Rodrigues, F. Nouar, U. H. Lee, K.-H. Cho, G. Maurin, J. A. C. Silva and C. Serre, Separation of Branched Alkanes Feeds by a Synergistic Action of Zeolite and Metal-Organic Framework, *Adv. Sci.*, 2022, **9**, 2201494.
[S15] L. Li, Z. Yang, Q. Wang, L. Yang, X. Suo, X. Cui and H. Xing, Efficient Separation of Di-branched Hexane from its Linear and Mono-branched Isomers via the Synergistic Molecular Sieving and Pore Shape-Matching Strategy, *Small*, 2025, **21**, 2412724.
[S16] L. Yu, X. Dong, Q. Gong, S. R. Acharya, Y. Lin, H. Wang, Y. Han, T. Thonhauser and J. Li, Splitting Mono- and Di-branched Alkane Isomers by a Robust Aluminum-Based Metal-Organic Framework Material with Optimal Pore Dimensions, *J. Am. Chem. Soc.*, 2020, **142**, 6925-6929.
[S17] P. S. Bárcia, A. Ferreira, J. Gascon, S. Aguado, J. A. C. Silva, A. E. Rodrigues and F. Kapteijn, Zeolite Beta membranes for the separation of hexane isomers, *Micropor. Mesopor. Mater.*, 2010, **128**, 194-202.
[S18] K. Yu, H. Xiao, J. Ding, S. Yan, K. Chi, Q. Gong, G. Qu, S. Sun, R. Zhou and W. Xing, Efficient separation of linear and mono-branched hexane isomers using large-area silicalite-1 membranes, *Sep. Purif. Technol.*, 2025, **354**, 129115.
[S19] T. Matsufuji, K. Watanabe, N. Nishiyama, Y. Egashira, M. Matsukata and K. Ueyama, Permeation of hexane isomers through an MFI membrane, *Ind. Eng. Chem. Res.*, 2000, **39**, 2434-2438.
[S20] C. L. Flanders, V. A. Tuan, R. D. Noble and J. L. Falconer, Separation of C_6 isomers by vapor permeation and pervaporation through ZSM-5 membranes, *J. Membr. Sci.*, 2000, **176**, 43-53.
[S21] B. H. Jeong, Y. Hasegawa, K. I. Sotowa, K. Kusakabe and S. Morooka, Vapor permeation properties of an NaY-type zeolite membrane for normal and branched hexanes, *Ind. Eng. Chem. Res.*, 2002, **41**, 1768-1773.
[S22] M. Woo, J. Choi and M. Tsapatsis, Poly(1-trimethylsilyl-1-propyne)/MFI composite membranes for butane separations, *Micropor. Mesopor. Mater.*, 2008, **110**, 330-338.
[S23] W. Yang, X. Yang, Y. Wang, R. Hou, Q. Gong and Y. Pan, Pervaporation separation of C_6 alkane isomers by Al-bttotb membrane, *J. Membr. Sci.*, 2022, **661**, 120916.
[S24] Y. Wang, Y. Ban, Z. Hu and W. Yang, Energy-efficient extraction of linear alkanes from various isomers using structured metal-organic framework membrane, *Nat. Commun.*, 2023, **14**, 6617.
[S25] J.R. Yu, P. P. Zhang, J. Y. Ding, Y. B. Liu, W. H. Lin, W. Shao, J. J. He, Q. Gong and M. Xue, UiO-66/PIM-1 Mixed-Matrix Membrane for Hexane Isomer Separation, *Inorg. Chem.,* 2024, **63**, 13031-13038.
